# Impact of Rearing Conditions on the Ambrosia Beetle’s Microbiome

**DOI:** 10.3390/life8040063

**Published:** 2018-12-13

**Authors:** Luis Arturo Ibarra-Juarez, Damaris Desgarennes, Mirna Vázquez-Rosas-Landa, Emanuel Villafan, Alexandro Alonso-Sánchez, Ofelia Ferrera-Rodríguez, Andrés Moya, Daniel Carrillo, Luisa Cruz, Gloria Carrión, Abel López-Buenfil, Clemente García-Avila, Enrique Ibarra-Laclette, Araceli Lamelas

**Affiliations:** 1 Red de Estudios Moleculares Avanzados, Instituto de Ecología A. C., Xalapa C.P. 91070, Mexico; luis.ibarra@inecol.mx (L.A.I.-J.); mirnavrl@gmail.com (M.V.-R.-L.); emanuel.villafan@inecol.mx (E.V.); alexandro.alonso@inecol.mx (A.A.-S.); ofelia.ferrera@inecol.mx (O.F.-R.); 2 Cátedras CONACyT. Instituto de Ecología, A. C., Carretera Antigua a Coatepec 351, Xalapa C.P. 91070, Mexico; 3 Red de Biodiversidad y Sistemática, Instituto de Ecología A. C., Xalapa C.P. 91070, Mexico; damaris.desgarennes@inecol.mx (D.D.); gloria.carrion@inecol.mx (G.C.); 4 Joint Unit of Research in Genomics and Health, Foundation for the Promotion of Health and Biomedical Research in the Valencian Community (FISABIO), 46010 Valencia, Spain; andres.moya@uv.es; 5 Institute for Integrative System Biology, University of Valencia-CSIC, 46010 Valencia, Spain; 6 Tropical Research and Education Center, University of Florida, Homestead, FL 33031, USA; dancar@ufl.edu (D.C.); luisafcruz@ufl.edu (L.C.); 7 Servicio Nacional de Sanidad, Inocuidad y Calidad Agroalimentaria, Unidad Integral de Diagnóstico, Servicios y Constatación, Tecámac, Estado de Mexico 55740, Mexico; abel.lopez@senasica.gob.mx (A.L.-B.); clemente.garcia@senasica.gob.mx (C.G.-A.)

**Keywords:** *Xyleborus* sp., metabolic capabilities, metagenomics, microbiota, fungus

## Abstract

Ambrosia beetles, along with termites and leafcutter ants, are the only fungus-farming lineages within the tree of life. Bacteria harbored by ambrosia beetles may play an essential role in the nutritional symbiotic interactions with their associated fungi; however, little is known about the impact of rearing conditions on the microbiota of ambrosia beetles. We have used culture-independent methods to explore the effect of rearing conditions on the microbiome associated with *Xyleborus affinis*, *Xyleborus bispinatus*, and *Xyleborus volvulus*, evaluating different media in laboratory-controlled conditions and comparing wild and laboratory conditions. Our results revealed that rearing conditions affected the fungal and bacterial microbiome structure and had a strong influence on bacterial metabolic capacities. We propose that the rearing conditions influence the ambrosia-associated fungal and bacterial communities. Furthermore, bacterial microbiome flexibility may help beetles adapt to different substrates.

## 1. Introduction

Every life form is intertwined with others through intimate and complex interactions; sometimes such connections are so permanent and relevant that they become symbioses. In a broad sense, symbioses refer to associations between organisms (often widely separated taxonomically) that occur during their evolutionary history, and confer an adaptive advantage to at least one of the organisms involved [1,2].

Among the most fascinating examples of insect-microbe symbioses are attine ants, termites, and ambrosia beetles, which evolved fungiculture enabling them to inhabit a wide range of niches [3,4]. Although these fungal farmer insects belong to divergent lineages, they all raise their brood on a fungal diet and have remarkably similar fungivorous niches, as well as homologous dominant bacterial constituents (comprising primarily the genera *Enterobacter*, *Rahnella*, and *Pseudomonas*) [5]. Furthermore, symbiotic bacterial communities participate in key processes such as protection, inter and intraspecific communication, mating initiation and reproduction, upgrading nutrient-poor diets, and assisting in the digestion of recalcitrant food components [1].

Unlike most fungus-cultivating termites, which acquire spores from the environment during the nest-founding stage, leafcutter ants and ambrosia beetles gather spores or mycelium from their natal nests before dispersing to establish new colonies [4]. It appears that in ambrosia beetles, independent origins of fungal farming have also led to the development of distinctive mycangia (specialized pockets to protect and transport the fungal spores), which differ in structure, location, size, and shape, as well as in the ability to secrete substances among species [6,7,8,9].

Fungal symbionts are essential for the survival of ambrosia beetles. Larvae and adults feed specifically on the symbiotic fungi cultivated on the plant-host xylem-sap wood [10]. Most ambrosia beetles (Coleoptera: Curculionidae: Scolytinae) are generalists, with a broad plant-host range, typically invading dead, sick, or stressed trees. For instance, *Xyleborus affinis*, *Xyleborus bispinatus*, and *Xyleborus volvulus* have been reported in several plant species [11,12]. However, few ambrosia beetle species are able to colonize healthy trees, including *Xyleborus glabratus* Eichhoff, which has invaded forests in the Eastern U.S., attacking several plant species in the Lauraceae [5].

Most of the knowledge gained regarding the ambrosia symbionts has been obtained through culturing techniques suitable for the growth of limited microbial species. Recently, studies using next-generation sequencing revealed the presence of diverse fungal and bacterial communities within the mycangia of ambrosia beetles [6,13,14].

Several studies suggest that the rearing substrate determines the composition of the microbial communities in the insect gut, along with other factors such as pH, host specificity, and life stage. Experimental evidence suggests that altering the insects’ diet could change the metabolic function of the gut communities and may also change the community structure [15,16].

Thus, insect’s microbiota could be consider “hidden” players that influence essential insect traits and that may affects nutrition and reproduction [17]. In ambrosia beetles, the role of the gut microbiota into the metabolic processes of their host is largely unknown. This becomes particularly relevant for some invasive ambrosia beetle species, with a wide hosts range but few reproductive hosts [18]. Fungal species associated with ambrosia beetles (cultured for nutritional purposes), can grow or infect reproductive and non-reproductive hosts, however, only in non-reproductive plant hosts beetles oviposition rates and/or the survivorship of progeny is low or absent [19]. Then, it is possible to hypothesize that beetles’ reproduction can be directly influenced by the host as the result of plant defense responses that, might affect the dynamic and diversity in the insect gut microbiota, or the growth of the symbiotic fungus inside the galleries [20].

Based on the previous knowledge there are some questions to be addressed if the host affects the microbiota of the gallery including the cultivated fungus, how does this influence the gut microbiota and/or their functions? Despite the increasing interest in ambrosia beetle’s research, studies on the gut microbiome and its influence on their insect host are still in an initial stage.

The present study compared the variation in abundance and diversity of bacterial and fungal communities in the head and abdomen of ambrosia beetles reared on media comprising sawdust of different plant species. In addition, the microbial communities associated with wild- and laboratory-reared beetles were compared. The function of the bacterial and fungal communities was predicted.

## 2. Materials and Methods

### 2.1. Beetle Collections

Specimens for laboratory rearing: *X. affinis* and *X. bispinatus* were collected using an ECOIAPAR trap [21], a handmade-trap used to catch the coffee berry borer. Traps were baited with 96% ethanol and placed in the field from 6 p.m. to 7 a.m. *X. affinis* was collected in Rivera Cupia, Chiapa de Corzo, Chiapas, Mexico and *X. bispinatus* from the Jaguaroundi Ecological Park Coatzacoalcos, Veracruz, Mexico.

Specimens of *X. volvulus* and *X. affinis* were collected from infested branches for wild-condition analyses. *X. volvulus* was collected from avocado (*Persea americana*) in Homestead, Florida, USA while *X. affinis* was collected from Chaca (*Bursera simaruba*) in Comoapa, San Andrés Tuxtla, Veracruz, México. Tree branches were placed inside emergence chambers according to Carrillo et al., 2012 [22].

Beetles were identified according to Wood (1982, 1986), using the key for Mexican *Xyleborus* genera [11,23,24,25], and stored at −20 °C.

### 2.2. Rearing Conditions

Analyses were performed of the microbiome and of the bacterial and fungal composition of head and abdomen of *X. bispinatus* and *X. bispinatus* reared on artificial media comprising two sawdust types (*Platanus mexicana* or *Persea schiedeana*) (Figure S1). In addition, the effect of the rearing conditions (wild vs. laboratory conditions) was examined by comparing the microbiome of wild *X. volvulus* and *X. bispinatus* females that had emerged from *Persea americana* and *Bursera simaruba* logs to the microbiome of *X. bispinatus* and *X. bispinatus* reared under laboratory conditions (Figure S1).

*X. affinis* and *X. bispinatus,* collected in ECOIAPAR traps, were reared on artificial media according to Menocal et al. (2017) [26], with some modifications. The media was prepared using 45 g of sawdust of either Coyo Avocado (*P. schiedeana*) or Mexican Sycamore (*P. mexicana*), agar (12 g), sucrose (6 g), casein (3 g), starch (3 g), yeast (3 g), Wesson’s salt mixture (0.6 g), wheat germ oil (1.5 mL), ethanol 96% (3 mL), and distilled water (400 mL). All ingredients were mixed and autoclaved for 30 min at 120 °C. Then 15 mL of medium was poured into 50 mL falcon tubes. The media were left to dry inside of a laminar flow hood overnight, and then tubes were closed and stored at −20 °C until use.

Before placing the females inside the tubes, the medium was poked with a dissecting needle to promote excavating activity. Tubes with females were placed inside an environmental chamber at 26 °C and 60% relative humidity. After 30 days, colonies were dissected, and six females were collected from each tube of the two types of rearing media.

### 2.3. DNA Library Construction

Six females per treatment were pooled and their surface disinfected with one minute washes of 90% ethanol, PBS and 0.1% tween20, with 70% ethanol, and H_2_O mQ. Head and abdomen were separately processed. DNA was extracted from the beetles F2 generation pools. Total DNA was extracted following the method described by Latorre et al. (1986) [27]. Ribosomal amplicons of the variable V3 region of the 16S gene were amplified using primers designed by Klindworth et al. (2003) [28]. Ribosomal amplicons of the 18S region were prepared to determine fungal diversity using primers SSUfungiF 5′-TGGAGGGCAAGTCTGGTG-3′ and SSUFungiR 5′-TCGGCATAGTTTATGGTTAAG-3′.

Illumina libraries were constructed by adding Nextera XT adapters (Illumina Inc., San Diego, CA, USA). Amplicon PCR and adapter-ligation PCR were performed using Kapa polymerase (Kapa HiFi Hotstart Ready Mix, cat. No. KK2602, Kapa Biosystems, Wilmington, MA, USA) following the manufacturer’s protocol and purified using 0.8X Agencourt Ampure XP cleaning beads (BeckmanCoulter, Brea, CA, USA, cat. No. A63881). Library concentration was quantified using a DNA HS kit (Invitrogen, Carlsbad, CA, USA, cat. No. Q32854) in a Qubit 2.0 fluorometer (Invitrogen, cat. No. Q32866). Adapter ligation and library size were evaluated using a Bioanalyzer (Agilent, Santa Clara, CA, USA, cat. No. G2953CA) DNA HS chip (Agilent, cat. No. 5067-4626). Libraries were diluted and pooled to an equimolar concentration to be denatured and loaded in a MiSeq (Illumina Inc., San Diego, CA, USA) using Reagent kit V3 (Illumina Inc. cat. No. 15067874).

Bacterial DNA sequencing was prepared in the Advanced Genomics Unit at LANGEBIO-CINVESTAV following the Illumina Inc. protocol (ref 15044223). 18S rDNA amplicon sequences were generated and sequenced by Macrogen, Rockville, MD, USA. The high throughput sequencing (HTS) datasets were deposited at the NCBI’s Sequence Read Archive with the number PRJNA490892.

### 2.4. Sequence Processing and OTUs Identification

Raw paired reads were filtered with PRINSEQ-lite 0.20.4 [29] to remove terminal nucleotides with quality score under and reads with mean quality score lower than 20 or a length under 50 nucleotides. Overlapping ends between the resulting high quality pairs were joined using the default parameters of the join_paired_ends.pl script [30,31]. Merged sequences were processed with MOTHUR v.1.25.0 [32] for the detection and removal of chimeric sequences; for the detection of the artifacts in all the bacterial sequences, the greengenes database was used as template (ftp://greengenes.microbio.me/greengenes_release/gg_13_5/gg_13_8_otus.tar.gz) whereas for fungal samples, the QIIME package release of SILVA database v128 [30,31] was used. (Table S1).

All the sequences of the same marker were concatenated into a single file after tagging each treatment with a unique barcode. A metadata mapping file was created with the information of each treatment, its barcode, and the linker primer used for amplification; the resulting file was verified with the validate_mapping_file.py script on QIIME package v. 1.9.0. [33]. Files containing all the sequences of each marker were filtered with the split_libraries.py script of QIIME package to remove sequences with more than two ambiguous nucleotides and regions composed by more than 10 homodimers. The OTU picking step was performed on these sequences using the pick_open_reference_otus.py script of QIIME package [34] by RDP method, clustering at 97% of homology using the greengenes database gg_13_8 for bacteria and clustering at 99% of homology using the SILVA database for fungi as reference. The resulting OTU table was converted into a tsv format with Python’s biom-format package v. 2.1.5 [35] in order to filter all the chloroplast, archaea, mitochondria, and cyanobacteria OTUs from the bacterial sequences; and Chloroplastida, Amoebozoa, Metazoa, SAR, and undefined eukaryotic OTUs from the fungal file. The fungal OTUs with a shallow taxonomic assignation were reassigned by BLASTN and confirmed by the phylogenetic tree built using an alignment of a subset of the SILVA database by FastTree v2.1.9 [36]. That was the case of NR.OTU105044 and EU011705.1.1709, reassigned as *Candida* for both OTUs.

### 2.5. Statistical Analyses of Identified OTUs

Rarefactions were produced from the filtered OTUs with the multiple_rarefactions.py script (QIIME package v 1.9.0) [parameters for 16S: −x 230,000 −m 10,000 −s 1,000 −n 5; parameters for 18S: −x 30,000 −m 1000 −s 100 −n 5]. Diversity indexes (Observed_OTUs, Shannon, and Simpson) were calculated, collated, and plotted with the alpha_diversity.py, collate_alpha.py, and make_rarefaction_plots.py scripts of QIIME package v. 1.9.0, respectively. Finally, other graphic summaries derived from the selected OTUs were created using vegan 2.4.0 [37], the RColorBrewer [38], and ggplot2 [39] packages of the R-project [40].

Differentially abundant bacterial and fungal OTUs were estimated with GFOLD V 1.1.4 [41], an algorithm designed to generate biologically meaningful rankings of differentially abundant taxa from samples without biological replicates. For this purpose, the normalized read counts of OTUs in each sample were used to estimate the fold changes (log2ratio); those with values log2fdc ≠ 0 and Gfold (0.01) ≠ 0, were considered as differentially abundant across all comparisons performed.

### 2.6. Metabolic Potential of the Fungal Communities

To gain insights into the metabolic potential of the fungi associated with the ambrosia beetles, we downloaded the closest genomes to the identified fungal OTUs from genebank (Tables S2 and S3), as well as those genomes with available annotations; otherwise, gene model prediction was performed (Table S4). In the latter case, repeats were first masked with RepeatMasker v4.0.6 [42] using the default Fungi database. The masked genomes were used as input for Augustus v3.1 [43], where different training sets and transcriptomic datasets were downloaded from SRA database [44] and assembled with Trinity v2.0.2 [45] (Table S5). Gene model predictions from Augustus, GeneMark-ES v4.32 [46] and SNAP [47], along with transcriptomic information, were used to generate a combined gene model prediction using Maker v3.01.02 [48]. At this step, a database of the proteomes of 11 Ascomycetes was used as homology evidence. For some genomes where gene model predictions were not satisfying, a previous annotation step was performed with Maker, using the complete UniProtKB/Swiss-Prot database as evidence of homology (Table S4, [49]). For some genomes, only the Augustus predictions were used (Table S4).

To obtain information from the functional contribution of each genus to the microbiota of the ambrosia beetles, the pan-genome was analyzed following get_homologues v3.0.9 pipeline [50]. The obtained core and flexible genes from all analyzed genomes (Table S5) were clustered by orthology using the OrthoMCL program [51]. Genus proteins were annotated using the KAAS-KEGG Automatic Annotation Server [52]. We identified the KEGG Orthologues specific to each genus by clustering analysis. We counted the number of KOs by functional category at the level L2 and L3 per fungal genus core proteins base on the KEGG database (Table S6).

A core gene set excluding all paralogues was built using Get homologues v3.0.9. These genes were concatenated and aligned using Mafft software [53]. The aligned sequences were then trimmed with Trimal [54] to remove non informative positions using the automatic trimming parameter suggested for maximum likelihood phylogenies. The phylogenetic reconstruction was performed using the 89 fungal core genes by FastTree v2.1.9 [36]. Support for nodes on the trees was assessed using 100 bootstrap replicates.

### 2.7. 18S rDNA Gene Phylogenetic Reconstruction

A subset of SILVA database containing all the sequences taxonomically related to the identified OTUs were used along with the Figure S13 phylogenetic reconstruction. All the sequences were aligned with the align_seqs.py script from QIIME package v1.9.0 [33], using the MAFFT algorithm and SILVA 104 database [30] as template. Aligned sequences were trimmed with Trimal [54] to remove non-informative positions using the automatic trimming parameter suggested for maximum likelihood phylogenies. Phylogenetic reconstruction was performed using FastTree v2.1.9 [36].

## 3. Results

### 3.1. Alpha Diversity Analysis of Bacterial and Fungal Microbiome

After filtering low-quality chimera sequences, and low frequency operational taxonomic units (OTUs) (<0.01%), sequencing of the 16S rDNA and 18S rDNA fragments from the beetle’s samples generated an average of 159,545.2 and 182,918.1 reads per sample, respectively (Table S1). The sequencing depth quality was confirmed by estimating the Shannon and Simpson alpha diversity index rarefaction curves, where all the curves reflected a saturated sampling (Figures S2 and S3).

The bacterial microbiome showed higher values of alpha diversity index and richness (OTUs number) than the fungal microbiome in all samples. The largest microbial richness was found on wild *X. bispinatus* collected from *B. simaruba* (X.Aff). In contrast, *X. bispinatus* reared on *P. schiedeana* (X.Bis.C) medium showed the smallest microbial diversity richness (Table 1).

Renyi’s community profiles revealed that wild *X. bispinatus* fungal and bacterial microbiomes were the most diverse and richest, respectively. In contrast, the fungal microbiome obtained from the head of *X. bispinatus* reared on *P. mexicana* and the bacterial microbiome of *X. bispinatus* head reared on *P. schiedeana* were the least rich. In addition, the fungal and bacterial microbiome of *X. bispinatus* reared on *P. schiedeana* showed the largest dominance. (Figure S4).

### 3.2. Core Microbiome of Beetles Reared under Laboratory and Wild Conditions

All the sequences were clustered in 720 bacterial and 34 fungal OTUs. The bacterial OTUs belong to six phyla: Actinobacteria (24 families), Bacteroidetes (10 families), Firmicutes (9 families), Proteobacteria (33 families), Tenericutes (1 family), and TM7 (1 family), with a mean frequency and standard deviation of 10.706 ± 8.564%, 0.051 ± 0.063%, 26.109 ± 19.649%, 0.597 ± 0.523%, 0.006 ± 0.013%, 61.682 ± 26.353%, 0.744 ± 2.281%, and 0.002 ± 0.006%, respectively.

To determine the core microbiome of the beetles reared under laboratory and wild conditions, we constructed a heatmap based on the relative abundance of the genera present in the fungal and bacterial microbiomes (Figures S5 and S6). We did not find any fungal genus shared by all the samples although *Talaromyces* was present in all samples with the exception of wild *X. volvulus*.

The core bacterial microbiome of all wild and lab samples included (average of relative abundance between samples ± SD): *Bacillus* (0.122 ± 0.086%), *Burkholderia* (12.791 ± 16.783%), *Enterococcus* (0.469 ± 0.330%), *Erwinia* (3.201 ± 3.456%), *Ochrobactrum* (1.470 ± 1.920%), *Propionibacterium* (0.078 ± 0,073%), *Pseudoxanthomonas* (0.100 ± 0.169%), *Sphingobacterium* (13.039 ± 12.448%), *Stenotrophomonas* (10.970 ± 15.323%), *Trabulsiella* (10.131 ± 18.825%). The genera *Janthinobacterium* (0.023 ± 0.008%), *Aeromicrobium* (0.026 ± 0.005%), *Comamonas* (0.111 ± 0.075%), *Azospirillum* (0.134 ± 0.089%), *Pimelobacter* (0.159 ± 0.081%), and *Entomoplasma* (0.443 ± 0.308%) were only present in wild samples, whereas there was no genus common to all lab-reared samples. The genus *Segniliparus* (3.265% ± 4.504%) was shared by *X. bispinatus* reared on both types of media but no genus was shared by different species reared on the same media.

### 3.3. Microbiome Structure of Beetles Reared under Laboratory Conditions

**Figure 1:**
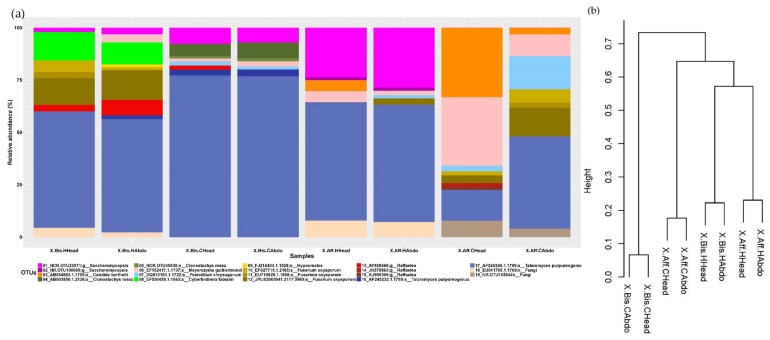
(**a**) Relative abundance of the fungal OTUs of *X. bispinatus* head reared on *P. mexicana* medium (X.Bis.HHead); Abdomen of *X. bispinatus* reared on *P. mexicana* medium (X.Bis.HAbdo). Head of *X. bispinatus* reared on *P. schiedeana* medium (X.Bis.CHead); Abdomen of *X. bispinatus* reared on *P. schiedeana* medium (X.Bis.CAbdo); Head of *X. affinis* reared on *P. mexicana* medium (X.Aff.HHead); Abdomen of *X. affinis* reared on *P. mexicana* medium(X.Aff.HAbdo); Head of *X. affinis* reared on *P. schiedeana* medium (X.Aff.CHead); Abdomen of *X. bispinatus* reared on *P. schiedeana* medium (X.Aff.CAbdo); Low-frequency OTUs were obtained by the sum of the OTUs with a relative frequency lower than 0.05%. (**b**) Dissimilarity cluster dendrogram of the binary matrix which corresponds at the presence/absence of bacterial OTUs of the laboratory reared samples.

**Figure 2:**
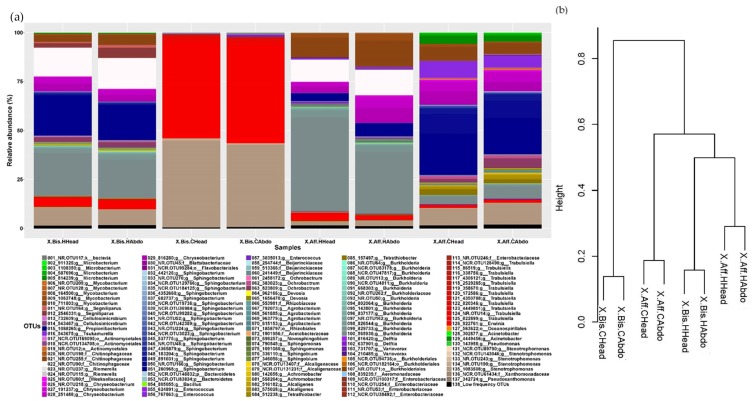
(**a**) Relative abundance of the bacterial OTUs in: Head of *X. bispinatus* reared on *P. mexicana* medium (X.Bis.HHead); Abdomen of *X. bispinatus* reared on *P. mexicana* medium (X.Bis.HAbdo). Head of *X. bispinatus* reared on *P. schiedeana* medium (X.Bis.CHead); Abdomen of *X. bispinatus* reared on *P. schiedeana* medium (X.Bis.CAbdo); Head of *X. affinis* reared on *P. mexicana* medium (X.Aff.HHead); Abdomen of *X. affinis* reared on *P. mexicana* medium (X.Aff.HAbdo); Head of *X. affinis* reared on *P. schiedeana* medium (X.Aff.CHead); Abdomen of *X. bispinatus* reared on *P. schiedeana* medium (X.Aff.CAbdo); Low-frequency OTUs: The sum of the OTUs with a relative frequency lower than 0.05%. (**b**) Dissimilarity cluster dendrogram of the binary matrix which corresponds to the presence/absence of bacterial OTUs of laboratory-reared samples.

To visualize the microbiome associated with the beetles reared on different artificial media, we constructed a stacked histogram (Figure 1a and Figure 2a). The most predominant fungal genus (average of relative abundance between samples ± SD) in *X. bispinatus* or *X. bispinatus* reared on *P. mexicana* or *P. schiedeana* medium was *Talaromyces*.

The analysis of the bacterial microbiome indicated that the most abundant genera in *X. bispinatus* reared on *P. mexicana* medium (X.Bis.HHead and X.Bis.HAbdo) were: *Chryseobacterium* (1.742 ± 0.198%) and *Stenotrophomonas* (1.727 ± 0.189%); in *X. bispinatus* reared on *P. schiedeana* medium (X.Bis.CHead and X.Bis.CAbdo) included: *Stenotrophomonas* (8.819 ± 0.377%) and *Trabulsiella* (4.302 ± 0.055%), in *X. bispinatus* reared on *P. mexicana* medium (X.Aff.HHead and X.Aff.HAbdo) were: *Burkholderia* (3.193 ± 0.873%) and *Mycobacterium* (2.968 ± 0.655%); and in *X. bispinatus* reared on *P. schiedeana* medium (X.Aff.CHead and X.Aff.CAbdo) were: *Tsukamurella* (7.329 ± 1.599%) and *Chryseobacterium* (2.933 ± 0.331%).

Based on the dissimilarity matrix calculated considering the presence and absence of bacterial and fungal OTUs, we constructed a dendrogram to establish a grouping pattern in both fungal and bacterial microbiomes. Interestingly, we observed the same pattern in both bacterial and fungal communities. The topology was characterized by clustering *X. bispinatus* and *X. bispinatus* reared on *P. mexicana* medium, while *X. bispinatus* reared on *P. schiedeana* medium grouped close to the *P. mexicana*-reared group, leaving *X. bispinatus* reared on *P. schiedeana* as an external group (Figure 1b and Figure 2b).

To detect differences between bacterial and fungal taxa, we applied the GFOLD algorithm [41] (Tables S7 and S8) and subsequently plotted the meaningful fold changes obtained to visualize potential patterns. We compared the fungal microbiome in abdomen vs. head of the same species reared on either *P. mexicana* or *P. schiedeana* (Figure S7). The head and abdomen of *X. bispinatus* reared on *P. mexicana* shared 11 fungal OTUs identified as follows: *Saccharomycopsis*, *Raffaelea*, *Candida*, *Fusarium oxysporum*, *Cyberlindnera fabianii*, *Talaromyces purpureogenus*, *Meyerozyma guilliermondii* and Hypocreales. When *X. bispinatus* was reared on *P. schiedeana* medium the head and abdomen shared seven of eight fungal OTUs identified as: *T. purpureogenus*, *Clonostachys rosea*, *Saccharomycopsis*, *T. purpureogenus*, and *Meyerozyma guilliermondii*. In the case of *X. bispinatus* reared on *P. mexicana* medium, the head and abdomen shared four of eight OTUs identified as *T. purpureogenus*, *Saccharomycopsis*, and *Candida* sp. The head and abdomen of *X. bispinatus* reared on *P. schiedeana* medium shared seven of nine fungal OTUs identified as: *Candida*, *M. guilliermondii*, *T. purpureogenus*, *F. oxysporum*, and *Penicillium chrysogenum*. All common OTUs exhibited significant differences in abundance in head and abdomen (Figure S7). (See Table S9 for the OTU code).

Relative abundance of bacterial OTUs differed between head and abdomen of the same beetle species reared on different media (Figure S8). For instance, the number of genera differing in abundance for *X. bispinatus* was 18 when it was reared on *P. mexicana* but only eight when reared on *P. schiedeana*. Likewise 28 genera differed in abundance in *X. bispinatus* reared on *P. mexicana* medium while 22 genera differed when it was reared on *P. schiedeana* medium (Figure S8).

The microbiome composition differed for the two beetle species reared on different media (Figure S9). In *X. bispinatus* reared on *P. mexicana* medium, we observed a large abundance of: *Candida*, *F. oxysporum*, *C. fabianii* and *Raffaelea*, with lower abundance of *P. chrysogenum* and *C. rosea*. While the microbiome of *X. bispinatus* reared on *P. mexicana* medium had a large abundance of *Saccharomycopsis* and *T. purpureogenus* but lower abundance of *F. oxysporum*, *M. guilliermondii*, *C. berthetii*, and *P. chrysogenum*, the latter also showed low abundance in *X. bispinatus* (Figure S9).

When the two beetle species were reared on *P. schiedeana* medium, the abundance of bacterial genera *Sphingobium*, *Burkholderia*, *Acinetobacter*, *Pseudomonas*, and *Mycobacterium* increased compared to the beetles reared on *P. mexicana*. However, when the beetles were reared on *P. mexicana* media, the genus *Stenotrophomonas* increased compared to those reared on *P. schiedeana* medium (Figure S10).

### 3.4. Microbiome Comparisons of Beetles Reared under Laboratory and Wild Conditions

**Figure 3:**
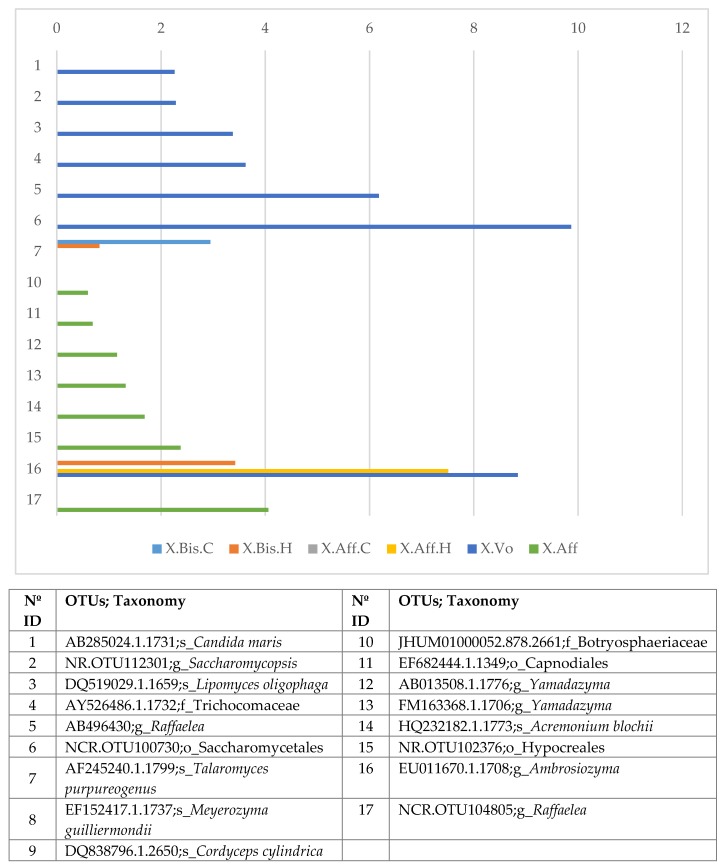
Differential abundance of fungal OTUs in wild- vs. lab-reared beetles. Wild-reared: *X. volvulus* reared on *P. americana* and *X. affinis* reared on *B. simaruba*; Lab-reared: *X. affinis* reared on *P. schiedeana* medium (Aff.C); *X. affinis* reared on *P. mexicana* medium (Aff.H); *X. bispinatus* reared on *P. schiedeana* medium (Bis.C); *X. bispinatus* reared on *P. mexicana* medium (Bis.H). Numbers on the y-axis represent the different functional categories listed in the table.

Using GFOLD algorithm, we analyzed the bacterial and fungal OTUs, and the corresponding bacterial genera present in the lab-reared and wild samples (Tables S10–S12). The bar chart with the fold change of the fungal OTUs (Figure 3) shows significant differences between laboratory-reared and wild *X. volvulus* or lab-reared and wild *X. bispinatus* OTUs, with larger abundance in wild *X. volvulus* and wild *X. bispinatus* than in laboratory-reared beetles. The OTUs with greatest abundance in wild *X. volvulus* were: *Candida maris*, *Saccharomycopsis*, *Lipomyces oligophage*, Trichocomaceae, *Raffaelea*, Saccharomycetales; whereas in wild *X. bispinatus* they were: Botryosphaeriaceae, *Cordyceps cylindrical*, Capnodiales, and *Yamadazyma*, *Acremonium blochii*, Hypocreales, *Ambrosiozyma*, and *Raffaelea*. In both cases *T. purpureogenus* and *M. guilliermondii* abundance was lower compared to the lab-reared beetles.

**Figure 4:**
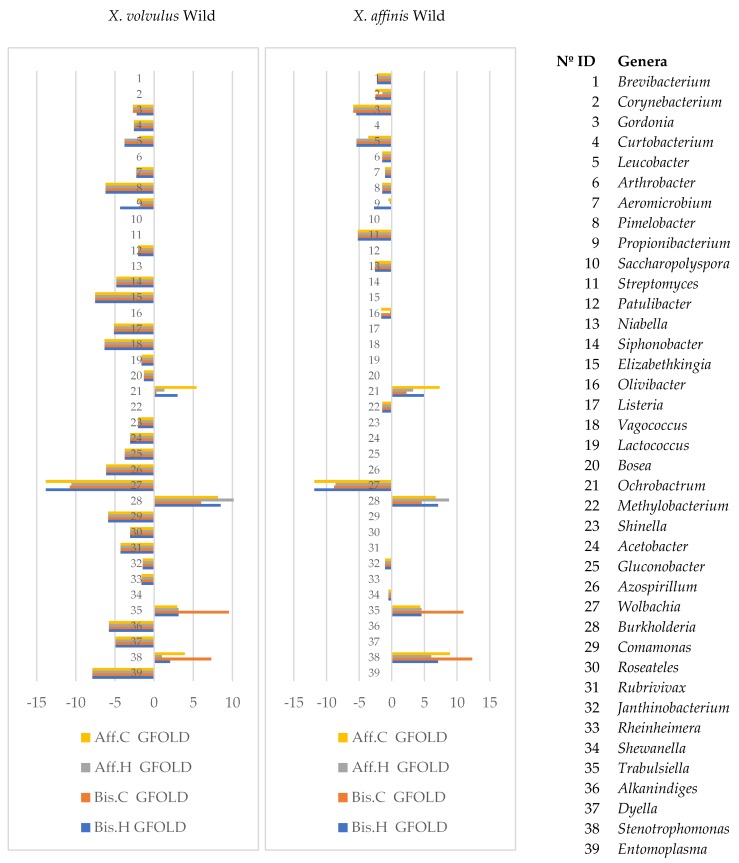
Differential abundance of bacterial genera in wild- vs. lab-reared beetles (log2fdc). *X. affinis* reared on *P. schiedeana* medium (Aff.C); *X. affinis* reared on *P. mexicana* medium (Aff.H); *X. bispinatus* reared on *P. schiedeana* medium (Bis.C); *X. bispinatus* reared on *P. mexicana* medium (Bis.H). Numbers on the *y*-axis represent the different functional categories listed in the table.

Analysis of the bacterial microbiome indicated that 661 OTUs had significant differences in abundance in at least one comparison between wild- and laboratory-reared samples, while 62 OTUs differed significantly in abundance in the 8 comparisons: *X. bispinatus* and *X. bispinatus* reared on *P. schiedeana* and reared on *P. mexicana* vs. *X. volvulus* reared on wild conditions and *X. bispinatus* and *X. bispinatus* reared on *P. schiedeana* and reared on *P. mexicana* vs. *X. bispinatus* reared on wild conditions (Aff.C, Bis.C, Aff.H, Bis.H vs. XVo and Aff.C, Bis.C, Aff.H, Bis.H vs. X.Aff) (Figure S11). These OTUs belonged mostly to the genera *Acinobacter*, *Aeromicrobium*, *Agrobacterium*, *Azospirillum*, *Enterococcus*, *Entomoplasma*, *Erwinia*, *Flavobacterium*, *Gordonia*, *Janthinobacterium*, *Luteimonas*, *Paenibacillus*, *Propionibacterium*, *Pseudomonas*, *Pseudoxanthomonas*, and *Wolbachia*. Statistical analysis of bacterial genera showed that 80 genera differed in abundance, 39 of which were significant across all comparisons between lab-reared and wild samples (Figure 4, Table S5). The genera *Gordonia*, *Leucobacter*, *Aeromicrobium*, *Pimelobacter*, *Propionibacterium*, *Wolbachia*, and *Janthinobacterium* had higher frequency in wild- than in lab-reared samples, while the genera *Ochrobactrum*, *Burkholderia*, *Trabulsiella*, and *Stenotrophomonas* were less frequent.

To determine whether the observed differences in the presence and abundance of bacterial and fungal OTUs between the microbiome of wild- and lab-rearing conditions affected community structure, we performed beta diversity analyses. For this, we calculated the UniFrac distance, and tested the significance by unweighted_unifrac and weighted_unifrac, drawing the PCoA with the QIIME package workflow.

**Figure 5:**
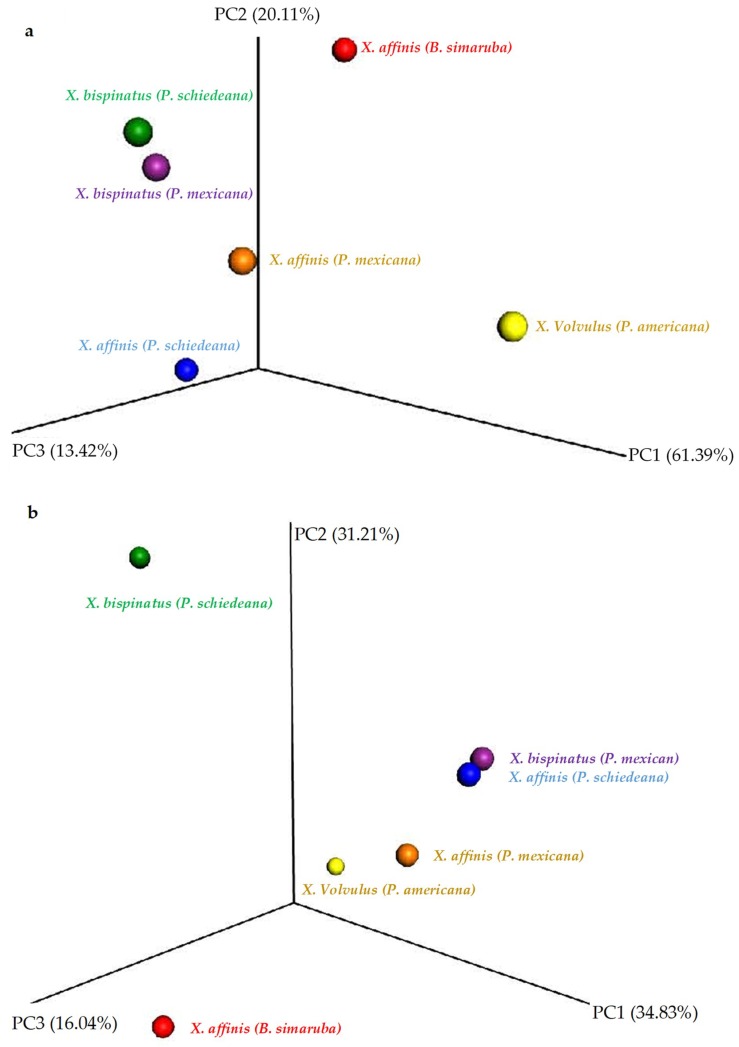
PCoA plot of the samples beta diversity Unweighted Unifrac of: (**a**) Fungal OTUs, (**b**) Bacterial OTUs.

The fungal communities were not clearly separated by either Unweighted UniFrac (Figure 5a) or Weighted UniFrac (Figure S12a), and inter-group partition was not significant. Contrarily, the PCoA Unweighted UniFrac analysis separated wild sample bacterial communities from the other samples by the 2nd PCoA axis (Figure 5b) and Weighted UniFrac by the 1st PCoA axis (Figure S12b). Moreover, the statistical test showed significant differences in the Unweight UniFrac analysis between X.Aff vs. X.Bis.H (*p*-value = 0.03), X.Aff.C vs. X.Bis.C (*p*-value = 0.03), X.Bis.C vs. X.Bis.H (*p*-value = 0.015).

### 3.5. Functional Metabolic Prediction of Fungal Genera

**Figure 6:**
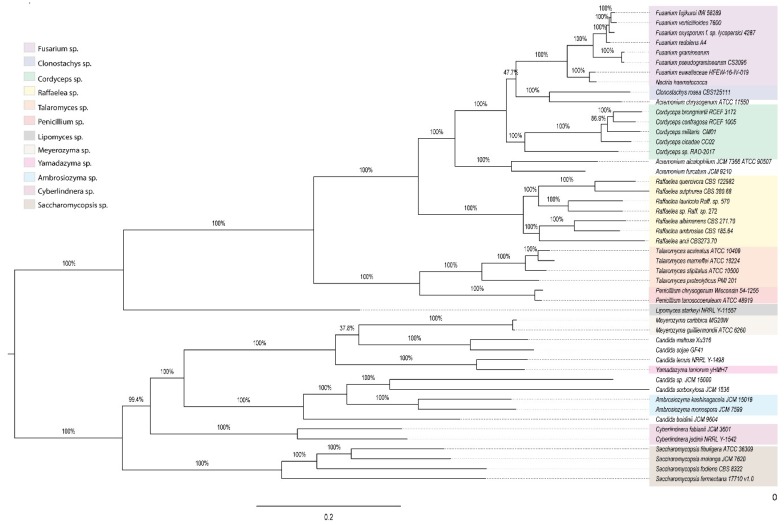
Phylogeny base for the 89 core genes of the fungal genomes (See Table S5), the colors represent the groups used to calculated the unique genes. The number on the branch indicate the bootstrap value.

The pan-genome of the fungi associated with ambrosia beetles comprised 106,474 orthologues predicted genes, 255 of which were shared by all fungal genomes analyzed; however, only 89 of these genes were single copy orthologue genes, constituting the core genes. The remaining 94,932 were cataloged as part of the flexible genome. With the 89 single-copy orthologue genes, we reconstructed the evolutionary relationships among the fungi associated with the ambrosia beetles (Figure 6, Table S13). Similarly to what we observed with 18S rRNA data (Figure S13), two main clades were generated; the first clustering filamentous fungi and the second clustering yeast species. The core phylogeny shows that besides *Acremonium*, *Clonostachys*, *Candida*, and *Yamadazyma*, the other fungal genera cluster as discrete groups.

To analyze the functional categories of the fungal microbiome per sample, metabolic categories were corrected for the frequency of every fungus in the sample. The dot-plot of the lab-reared microbiomes showed that “Signal transduction (8.78 ± 0.31%)” and “Folding sorting and degradation (11.17 ± 0.20%)“, were the categories with greatest differences in frequency. (Figure S14). Deeper analysis did not show any differences between head and abdomen except for X.Aff.CHead and X.Aff.CAbdo. In addition, the fungal microbiome of *X. bispinatus* reared on *P. mexicana* medium had a greater frequency for “Cell growth and death” and “Replication” than *X. bispinatus* reared on *P. schiedeana*, while frequency was lower on “Xenobiotics biodegradation and metabolism” and “Unclassified:Metabolism”. In regard to *X. bispinatus* reared on *P. mexicana* medium the metabolic categories “Translation”, “Carbohydrate metabolism”, and “Enzyme families” showed greater frequency than *X. bispinatus* reared on *P. schiedeana*, and lower frequency for “Replication and repair” and “Metabolism of terpenoids and polyketides” than *X. bispinatus* reared on *P. schiedeana*. The dot-plot graph did not show a rearing medium effect in the metabolic category frequencies. (Figure S14).

We performed a similar analysis comparing wild and laboratory-reared beetles (Figure S15). The functional categories of the beetle’s fungal microbiomes with a larger frequency range were: “Signal transduction (9.04 ± 0.86%)”, “Folding, sorting, and degradation (9.73 ± 0.54%)”, “Replication and repair (7.38 ± 0.52%)”, “Transcription (8.39 ± 0.50%)”, “Translation (11.23 ± 0.43%)”, “Carbohydrate metabolism (7.45 ± 0.40%)”, “Nucleotide metabolism (4.50 ± 0.28%)”, “Energy metabolism (3.35 ± 0.27%)”, “Lipid metabolism (4.40 ± 0.25%)”, and “Amino acid metabolism (7.28 ± 0.22%)”. Wild-reared *X. volvulus* and *X. bispinatus* showed the highest and lowest frequencies in the different functional categories, where the intermedium frequencies were exhibited by the lab-reared samples (Figure S15).

Analysis of presence and absence of fungal microbiome KOs per sample showed that almost all the microbiomes were equally able to develop the different metabolic categories. We found 56 unique KOs in the fungal microbiome of wild-reared beetles, distributed in the following functional categories: “Enzyme families (15 KOs)”, “Unclassified: Genetic information processing (30 KOs)”, “Unclassified: Signaling and cellular processes (4 KOs)”. In the fungal microbiome of laboratory-reared beetles we found 223 unique KOs distributed in the functional categories: “Enzyme families (89 KOs)”, “Unclassified: Genetic information processing (116 KOs)”, and “Unclassified: Signaling and cellular processes (18 KOs)”.

### 3.6. Bacterial Functional Categories between Laboratory-Reared Beetles

We found 5443 KEGG Orthology (KO), predicted by PICRUST software. To establish a grouping pattern based on microbiome functionality, we constructed dendrograms (Figure S16). The resulting pattern agreed with the dendrogram of the presence and absence of bacterial OTUs, where *X. bispinatus* and *X. bispinatus* reared on *P. mexicana* medium, clustered with of *X. bispinatus* reared on *P. schiedeana* medium, leaving *X. bispinatus* reared on *P. schiedeana* medium as the external group.

To illustrate the metabolic differences between the body parts of the beetles we plotted the fold change differences between head and abdomen of *X. bispinatus* and *X. bispinatus* reared on different media (Figure S17). The samples of *X. bispinatus* reared on the two artificial media and *X. bispinatus* reared on *P. mexicana* medium, showed the same functional pattern. The principal functional categories related with “Metabolism” were more abundant in abdomens than in heads, while the categories related to “Cellular Processes” were more abundant in heads than in abdomens. In *X. bispinatus* reared on *P. schiedeana* medium, the functional categories that increased in abdomens compared to heads were principally regarding to “Cellular Processes” and “Genetic Information Processing”. In contrast, the categories related to “Metabolism“ decreased in abdomens compared to heads (Figure S17).

To visualize the effect of the media on the metabolic functions of the bacterial microbiome of *X. bispinatus* and *X. bispinatus*, we performed the GFOLD analysis, represented with bar charts using the KO’s categories: Bis.H.Head vs. Bis.C.Head, Bis.H.Abdo vs. Bis.C.Abdo, Aff.H.Head vs. Aff.C.Head and Aff.H.Abdo vs. Aff.C.Abdo (Figure S18). The analysis showed that the effect of the rearing medium depended on the ambrosia beetle species. The functional categories with greater abundance in the insect samples reared on *P. mexicana* medium were “Transcription” and “Xenobiotics biodegradation and metabolism” (Figure S18).

### 3.7. Bacterial Functional Categories in Wild- Versus Laboratory-Reared Beetles

**Figure 7:**
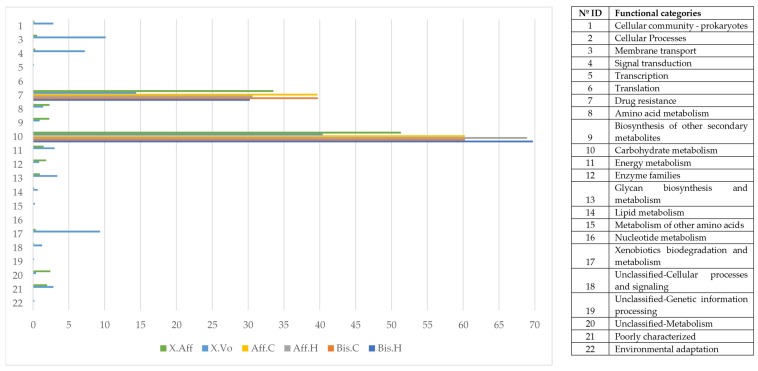
Functional categories of the bacterial microbiome under different rearing conditions. *X. affinis* reared on *P. schiedeana* medium (Aff.C); *X. affinis* reared on *P. mexicana* medium (Aff.H); *X. bispinatus* reared on *P. schiedeana* medium (Bis.C); *X. bispinatus* reared on *P. mexicana* medium (Bis.H). Numbers on the y-axis represent the different functional categories listed in the table.

The predicted metabolic functional categories of the bacterial microbiome of wild and laboratory beetles were compared using GFOLD statistical analysis (Tables S14–S16). Although the abundance of 5348 KOs was significantly different, when all the samples were compared (lab-reared vs. wild) only 74 were significantly different (Figure S19 and Table S17). We grouped all the fold changes of KOs by L2 functional categories of KEGG (Figure 7).

The metabolic functional categories over-represented in the microbiome of the wild samples were: “Environmental Information Processing ”, “Metabolism”, “Cellular Processes”, “Genetic Information Processing”, and “Organismal Systems”. The category that exhibited less abundance in the wild samples was “Drug resistance” (−1.74 ± 0.67 fold change).

The functional category “Membrane transport” was composed of the sub-categories “Secretion system” and “Bacterial secretion system” being the most abundant with 50.49 ± 2.089%, and 23.41 ± 1.59%, respectively. The category “Energy metabolism”, consisted of several sub-categories, with “Methane metabolism” being the most abundant with 77.94 ± 2.098%.

## 4. Discussion

Insect diet affects the composition of the gut bacterial community [55,56]. In this study, we tested the effect of the rearing medium on the fungal and bacterial microbiome to determine how nutritional resources in the medium influence the microbiome structure of ambrosia beetles.

The number of OTUs estimated in our study ranged from 6 to 15 for fungi and from 64 to 312 for bacteria. The fungal OTU values were lower than those previously published by Kostovcik et al. (2015) [6], who estimated 15 OTUs per beetle species. In contrast, the bacterial microbiome was higher than that estimated by Hulcr et al. (2007) [13], who reported a range of 1 to 128 bacterial OTUs per beetle species. This discrepancy could be due to differences in the sequencing platforms used between studies. The alpha diversity, as well as the number of OTUs, observed per sample, depended on the rearing medium; however, comparison of rearing conditions (wild vs. laboratory) failed to detect any trends (neither lower nor higher microbiome biodiversity).

The impact of rearing conditions on the fungal and bacterial microbiome was evaluated by a fold change analysis (GOLD study) on four different comparisons:

(a) *Head vs. abdomen of the same species reared on the same medium.* Although the microbiomes in the head and abdomen were similar, the relative frequency of some OTUs, bacterial genera, and bacterial metabolic functions varied among samples. Changes in the microbiome structure depended on the rearing medium and the beetle species. In both cases, the differences in fungal and bacterial microbiome could be explained by the environmental conditions. The fungal and bacterial microbiomes in the head may be exposed to different pH, oxygen levels, and nutrient availability compared to the abdomen. Moreover, the role of the microbiome varies in the different parts of the beetle body; the head encloses the mycangia, which act as a receptacle to preserve microorganisms while the abdomen (gut) contains most of the beetle’s digestive system. The principal functions of the bacterial microbiome in the head were found to be related to communication between microbiomes: “Cellular Processes”, “Cellular community—prokaryotes”, “Cell motility”, “Signal transduction”, “Membrane transport”, “Transport and catabolism”, “Transcription”, “Drug resistance”, and “Biosynthesis of other secondary metabolites”, while in the abdomen the functions were related to nutrition: “Cell growth and death”, “Folding, sorting and degradation”, “Amino acid metabolism”, “Infectious diseases”, “Enzyme families”, “Energy metabolism”, “Carbohydrate metabolism”, “Xenobiotics biodegradation and metabolism”, “Nucleotide metabolism”, “Metabolism of terpenoids and polyketides”, “Metabolism of other amino acids”, “Metabolism of cofactors and vitamins”, and “Lipid metabolism”. These results are consistent with the functions of the head and abdomen in beetles. Surprisingly, the study of the functional abilities of the fungal genomes did not differ greatly between samples.
(b) *Same species reared on different media.* The microbiome structure varied with the media and the beetle species, increasing the abundance of the bacterial genera *Sphingobium*, *Burkholderia*, *Acinetobacter*, *Pseudomonas*, and *Mycobacterium* in *P. schiedeana* compared to the beetles reared on *P. mexicana*. The metabolic categories “Transcription” and “Xenobiotics biodegradation and metabolism” increased in *P. mexicana* rearing medium, the fungal microbiome metabolic capabilities remained unchanged. The genera *Acinetobacter* and *Pseudomonas* were linked with phenolic glycoside metabolism in gypsy moth [17] and terpenes metabolism in *Dendroctonus ponderosa* [57]. Phenolic and terpenoids compounds they are part of the plant defenses and has been describe the role of the bark beetle’s microbiome in the detoxification of these molecules [17]. The increment of these bacterial genera in *P. schiedeana* could be explain for a modification of the levels of these components in the rearing medium. This hypothesis should be verified.
(c) *Wild vs. laboratory rearing conditions.* All the laboratory samples exhibited large abundance on *T. purpureogenus* and *M. guilliermondii*. Regarding the bacterial microbiome, 62 OTUs were more abundant in the wild than in lab-reared samples. The genera *Gordonia*, *Leucobacter*, *Aeromicrobium*, *Pimelobacter*, *Propionibacterium*, *Wolbachia*, and *Janthinobacterium* were more abundant in wild than in laboratory samples, while *Ochrobactrum*, *Burkholderia*, *Trabulsiella*, and *Stenotrophomonas* were more abundant in laboratory than in wild beetles. All these genera have been found in other insect microbiomes [3,13,17,58]. The fungal metabolic categories did not differ between the microbiomes associated to the different rearing conditions. The metabolic categories with greater abundance in wild than laboratory conditions were related to: “Basic cellular functions”: Transcription and translation, cellular processes, and signaling. “Metabolism”: Obtaining energy and production of basic components. “Defense”: Metabolism of secondary metabolites, Degradation of xenobiotics, Signal transduction (Bacterial toxins and Two-component system). “Communication”: Cell communication, Membrane, and Plant-pathogen interaction.

Under wild-rearing conditions biotic and abiotic factors are uncontrolled, making competition for survival more complicated than under lab-rearing conditions. The survival strategy adopted by most organisms in variable environments involves increased adaptability to environmental fluctuations through heightened functional repertoire derived from increased gene diversity [59]. Prokaryotic organisms exhibit larger effective population sizes and faster replication rates than eukaryotic organisms [60]. For this reason, one strategy displayed by eukaryotic organisms is to modify their microbiota, which helps them to adapt to a variable environment [61]. Although there is no clear increase in microbial diversity in wild individuals, an increase in the diversity of metabolic functions can be observed, including transcription, replication, energy acquisition from more diverse sources, defense and communication, all metabolic functions that make the beetles more suitable to wild conditions.

Microbiomes exist in all ecosystems and are composed of diverse microbial communities. It has been proven that perturbation to microbiomes could bring about undesirable phenotypes in the hosts, resulting in diseases and disorders, and disturbs the balance of the associated ecosystems, however, these microbiomes can be modified [62]. Engineering of microbiomes can be used to modify structures of the microbiota and restore ecological balance or provide desirable specific features. Microbiome engineering has been employed for improving human health and agricultural productivity [62], and it can be used as a control strategy against the phytopathogenic fungus associated with ambrosia beetles. Success on the engineering of microbiomes depends on deep knowledge of microbiomes, and this is the first report about the impact of rearing conditions on the ambrosia beetle’s microbiome. Based on these results, future studies must be performed using other biochemical and bioinformatics strategies which reveal more accurate results.

Fungal microbiome functionality was not greatly altered between species or between rearing conditions, while the bacterial microbiome showed a drastic metabolic change, especially when wild and laboratory rearing conditions were compared. There was an increase in functional capabilities exhibited by wild samples, including Cellular processes, Metabolism, Defense, and Communication compared to laboratory samples, suggesting enhanced beetle adaptability to more diverse media.

## Figures and Tables

**Table 1:** Estimation of alpha diversity index and richness (number of OTUs) of bacterial and fungal microbiomes.

		Bacterial Samples			Fungal Samples		
ID Samples	Specie Name	Observed OTUs	Shannon Index	Simpson Index	Observed OTUs	Shannon Index	Simpson Index
X.Aff.CAbdo	*X. affinis*	199	4.596	0.925	8	2.405	0.744
X.Aff.CHead	*X. affinis*	180	4.347	0.912	9	2.368	0.752
X.Aff.HAbdo	*X. affinis*	158	3.713	0.850	7	1.714	0.597
X.Aff.HHead	*X. affinis*	169	3.231	0.743	6	1.783	0.614
X.Bis.CAbdo	*X. bispinatus*	73	1.813	0.597	7	1.314	0.397
X.Bis.CHead	*X. bispinatus*	64	1.728	0.591	8	1.343	0.393
X.Bis.HAbdo	*X. bispinatus*	178	4.034	0.888	10	2.232	0.667
X.Bis.HHead	*X. bispinatus*	178	3.904	0.874	8	2.085	0.648
X.Aff	*X. affinis*	312	3.447	0.744	15	2.438	0.669
X.Vo	*X. volvulus*	192	3.631	0.758	9	1.984	0.588

## Supplementary Materials

The following are available online at http://www.mdpi.com/2075-1729/8/4/63/s1, Figure S1: Procurement sampling scheme, Figure S2: Rarefaction curves of pyrosequenced fungal OTUs, Figure S3: Rarefaction curves of pyrosequenced bacterial OTUs, Figure S4: Renyi profiles of the fungal microbiome and bacterial microbiome, Figure S5: Relative abundance of the fungal taxa by sample, Figure S6: Relative abundance of the bacterial genera by sample, Figure S7: Relative abundance of the fungal OTUs significantly different in the abdomen or the head of *X. bispinatus* and *X. affinis* reared on the different artificial media, Figure S8: Relative abundance of bacterial genera differentially present in the abdomen compared to head of the adult females of *X. bispinatus* and *X. affinis* reared on different artificial media, Figure S9: Relative abundance of the fungal OTUs differentially present in the abdomen and head of *X. affinis* and *X. bispinatus* comparing rearing conditions, Figure S10: Relative abundance of the bacterial genera significantly different in the abdomen and the head of *X. affinis* and *X. bispinatus* comparing rearing conditions, Figure S11: Fold changes (GFOLD (0.01)) of the OTUs with significantly larger abundance in wild beetles compared to the lab-reared beetles, Figure S12: PCoA plot of the samples beta diversity of Weighted Unifrac, Figure S13: Phylogenetic tree of OTUs and reference sequences, Figure S14. Dot-plot of the functional category frequencies of the beetle’s fungal microbiome under laboratory-reared conditions, Figure S15. Dot-plot of the functional category frequencies of the fungal microbiome of the samples under study, Figure S16: Cluster dendrogram of dissimilarity matrix of the abundance of KOs, Figure S17: Relative frequency of the functional categories with differences in abundance between head and abdomen of the beetles reared under lab condition, Figure S18: Relative abundance of the functional categories with differences in abundance between *X. bispinatus* and *X. affinis* reared under lab conditions., Figure S19: Change fold significate different of the KO’s between wild and reared lab condition beetles., Table S1: List of samples and associated metadata used in this study, Table S2: Fungal OTUs. OTUs IDs and corresponding taxonomy, Table S3: Overview of the genome data used for the fungal community functional analysis, Table S4: Information used under Augustus gene model prediction, Table S5: Core and flexible genome metrics, Table S6: Annotation with KAAS-KEGG using KO identifiers, Table S7: Fold change of the significat fungal OTUs belonging to the head and abdomen of the beetles reared on artificial media, Table S8: Fold change of bacterial genera belonging to the abdomen and head of the beetles reared on artificial media, Table S9: Bacterial OTUs. OTUs IDs and its corresponding taxonomy, Table S10: Fold change of bacterial OTUs of the wild and artificially reared beetles, Table S11: Fold change of bacterial genera from the wild and artificially reared beetles, Table S12: Fold change of fungal OTUs of wild and artificially reared beetles, Table S13: Relative frequencies of significate different of the KO’s between wild and reared lab condition beetles, Table S14: Fold change of bacterial KOs belonging to the beetles reared on artificial media, Table S15. Fold change of bacterial KOs from wild and artificially reared beetles. Table S16. Relative frequencies of significate different of the KO’s between wild and reared lab condition beetles. Table S17. Functional categories of the KEGG Orthology (KO’s) with significate abundance difference between wild and reared lab conditions beetles.
