# Supplementary material for: Impact of Rearing Conditions on the Ambrosia Beetle’s Microbiome
Source: Life (Basel). 2018 Dec 13;8(4):63. doi: 10.3390/life8040063 (PMC6316638; doi:10.3390/life8040063)
Supplement: Supplementary file 1 [file life-08-00063-s001.zip › Supp.figures_07-11-18.docx]

**a)**

P


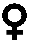


F1


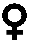


F2


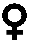


*Persea schiedeana*


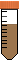


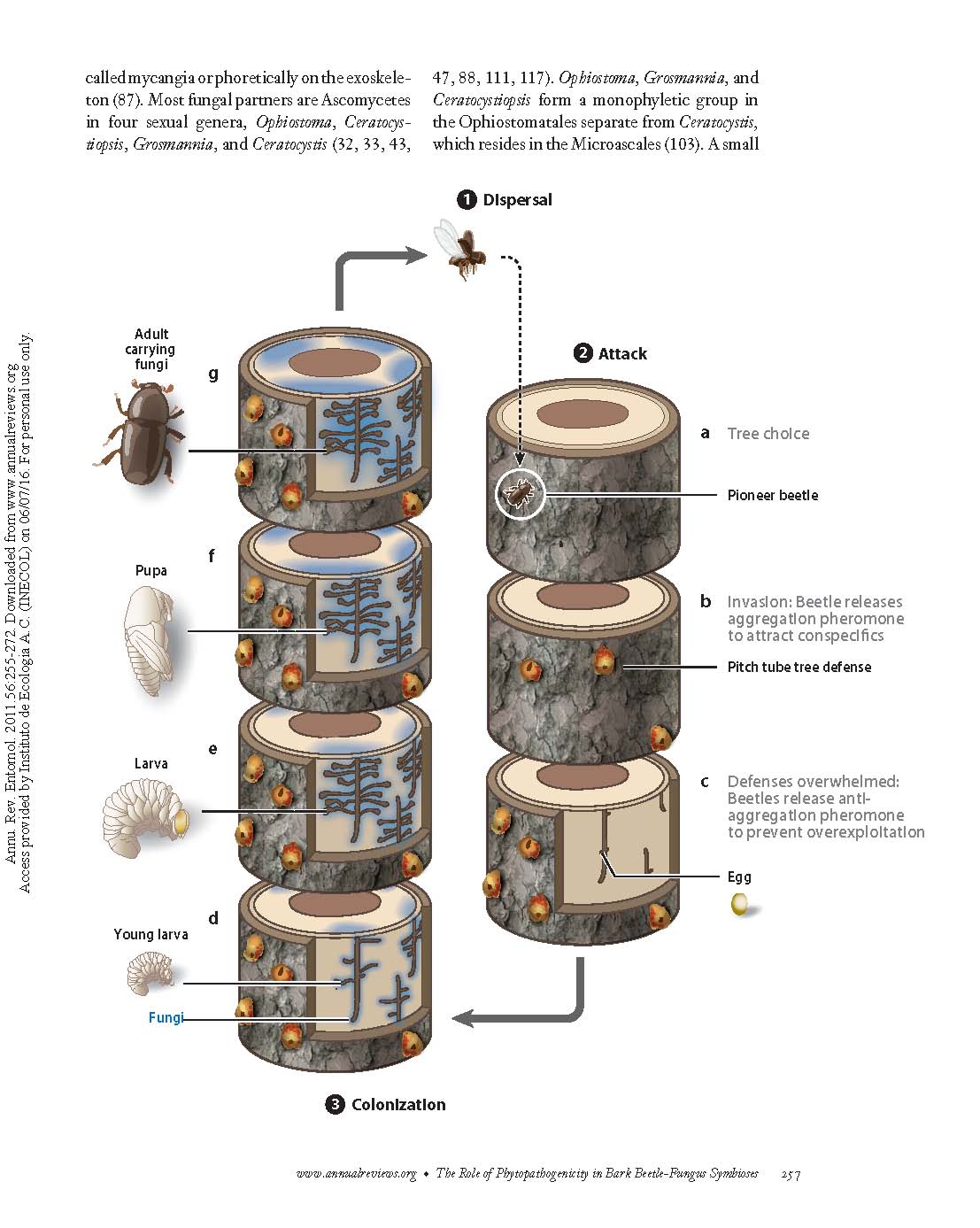


XA


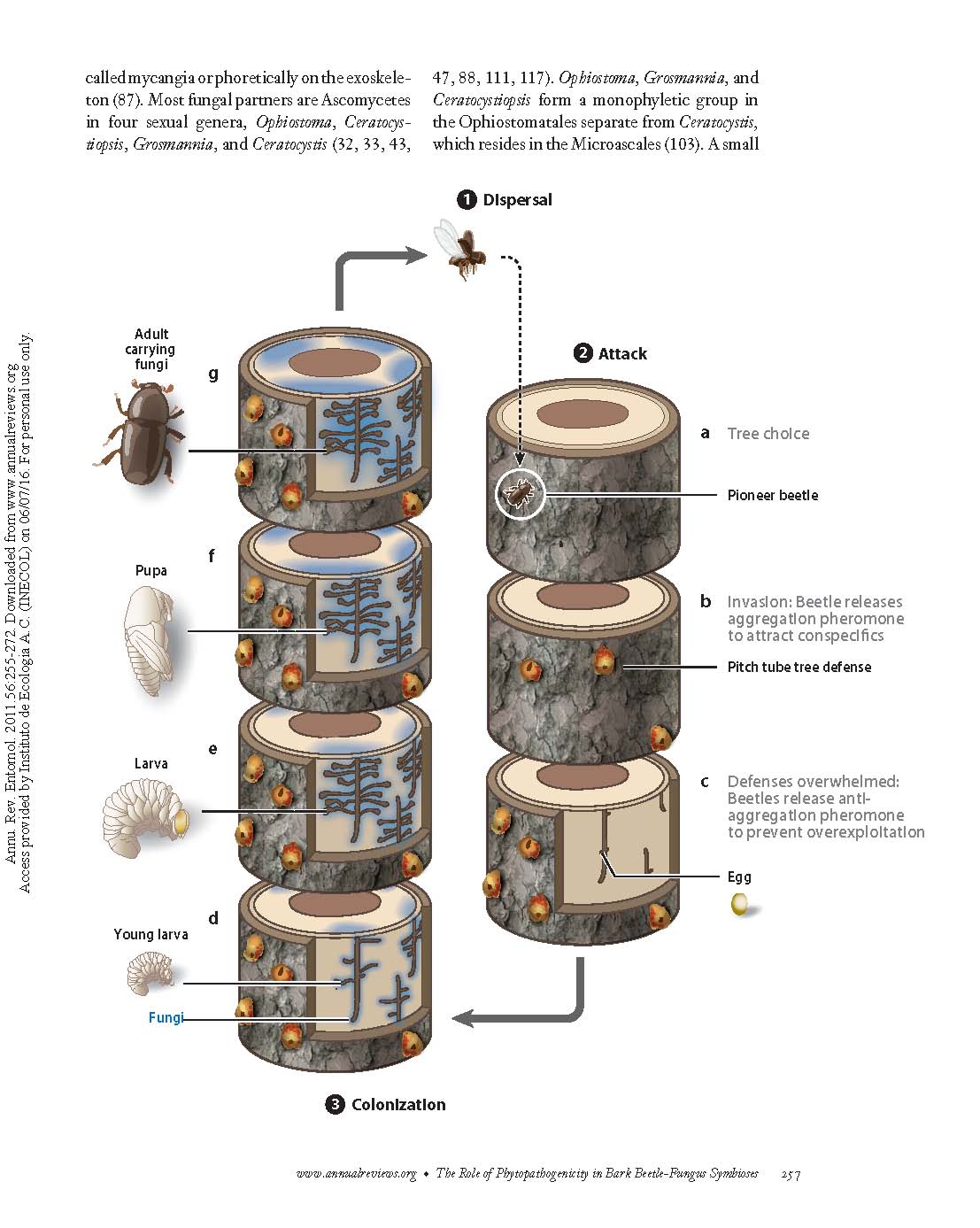


XA

*X. affinis*

*Platanus mexicana*


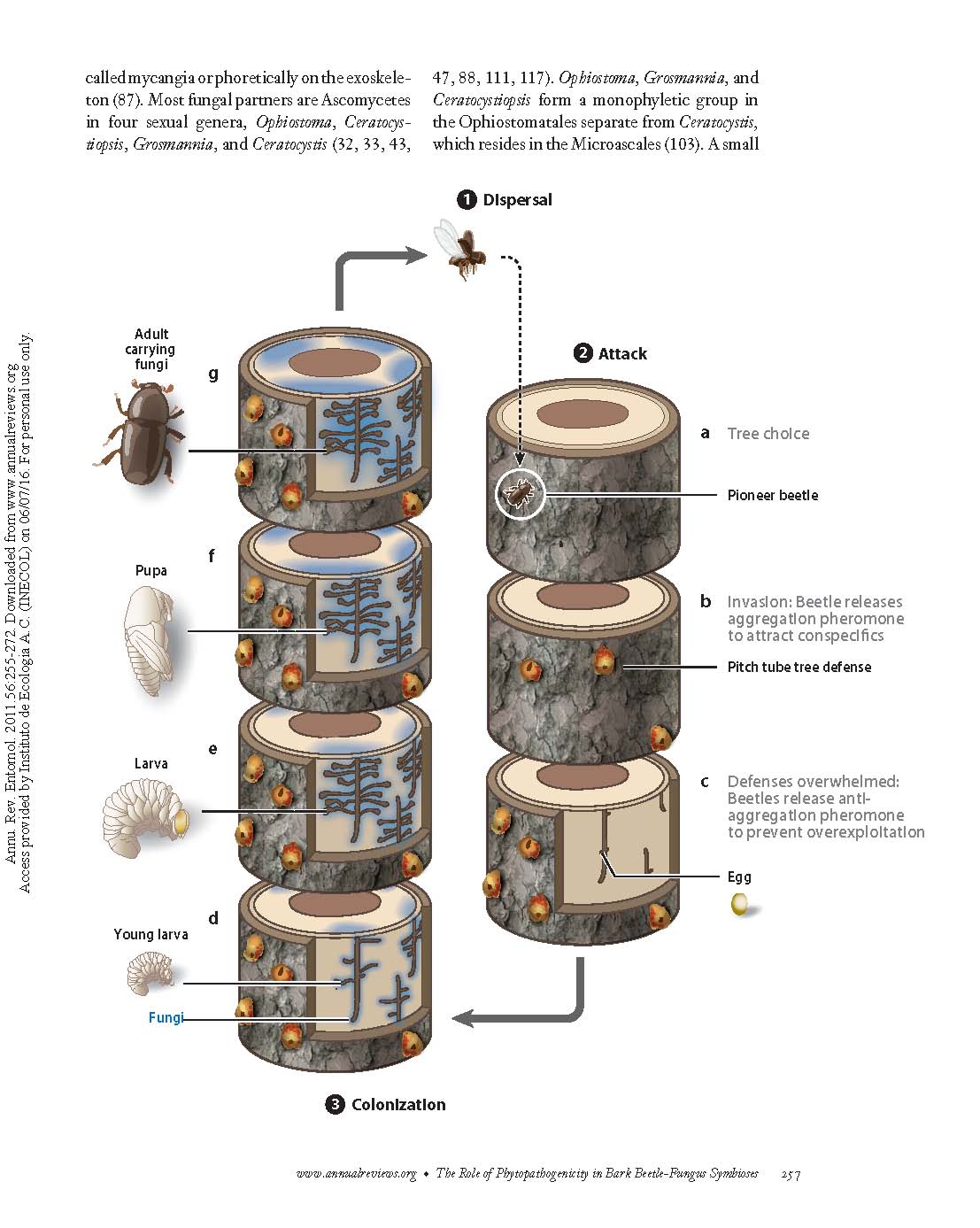


XA


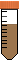


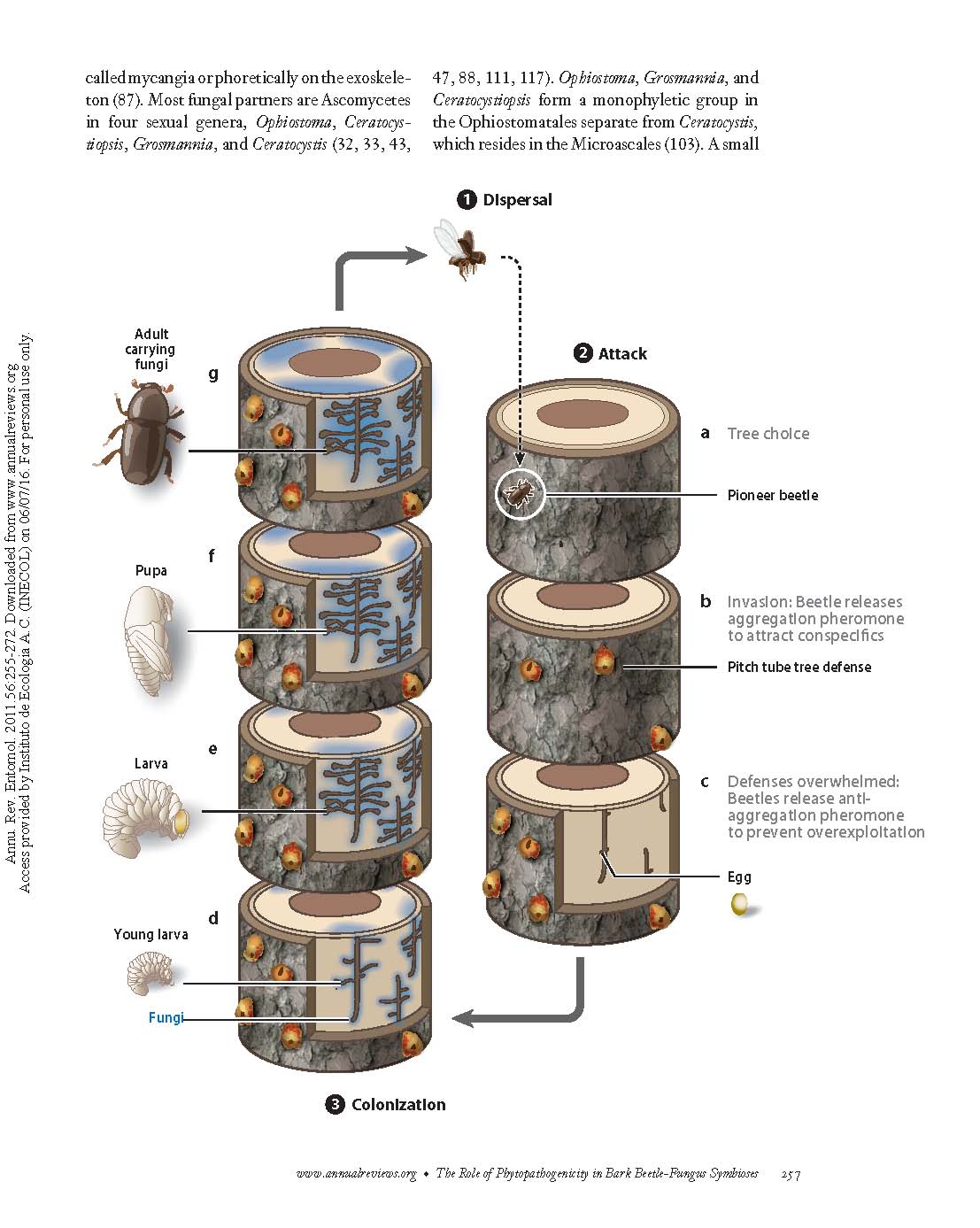


XA


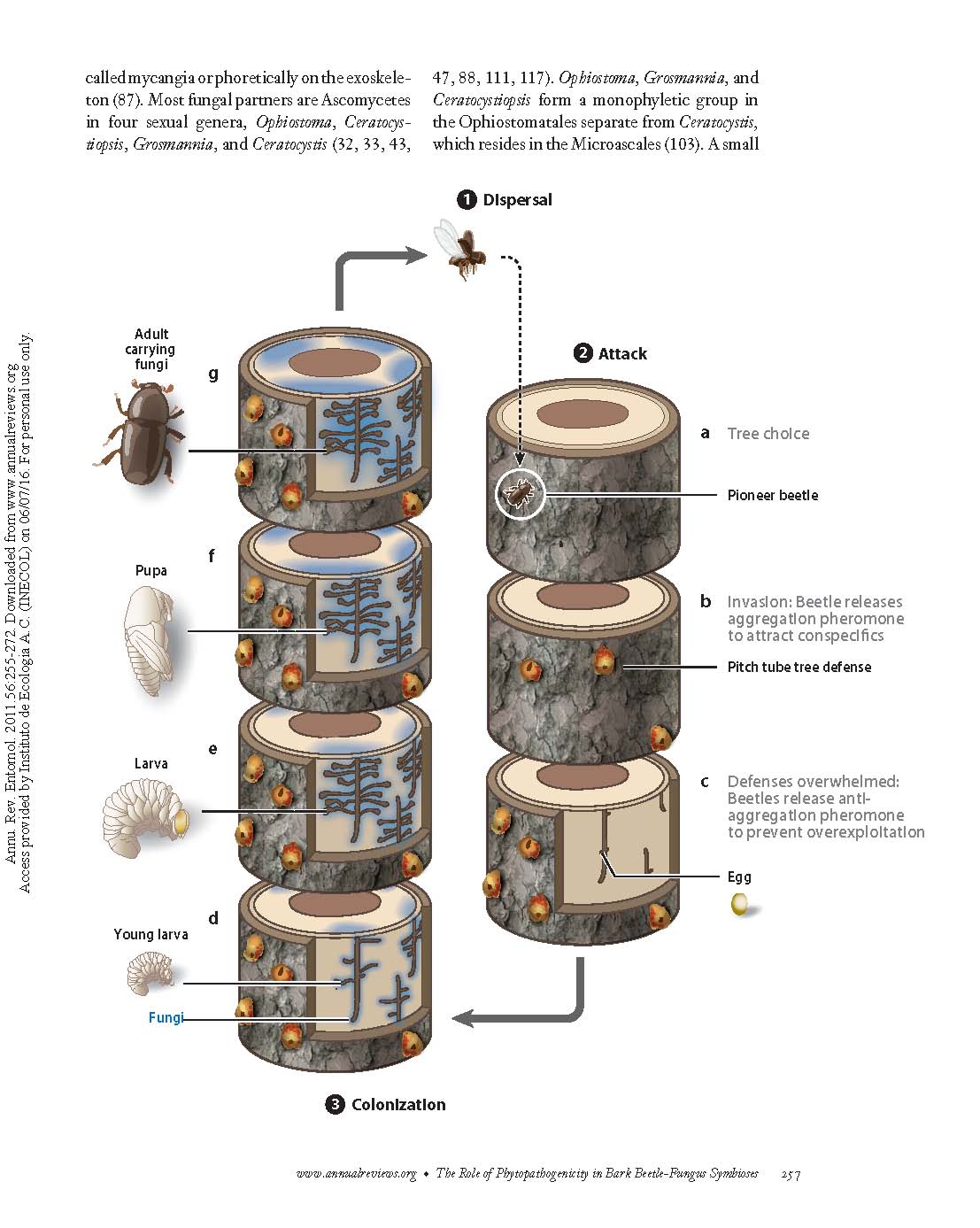


XA

*Persea schiedeana*


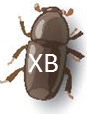

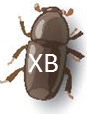

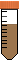


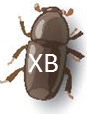


*X. bispinatus*

*Platanus mexicana*


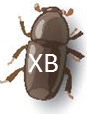

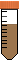

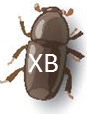


**b)**

F1


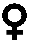


P


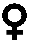


*X. affinis*

*Bursera simaruba*


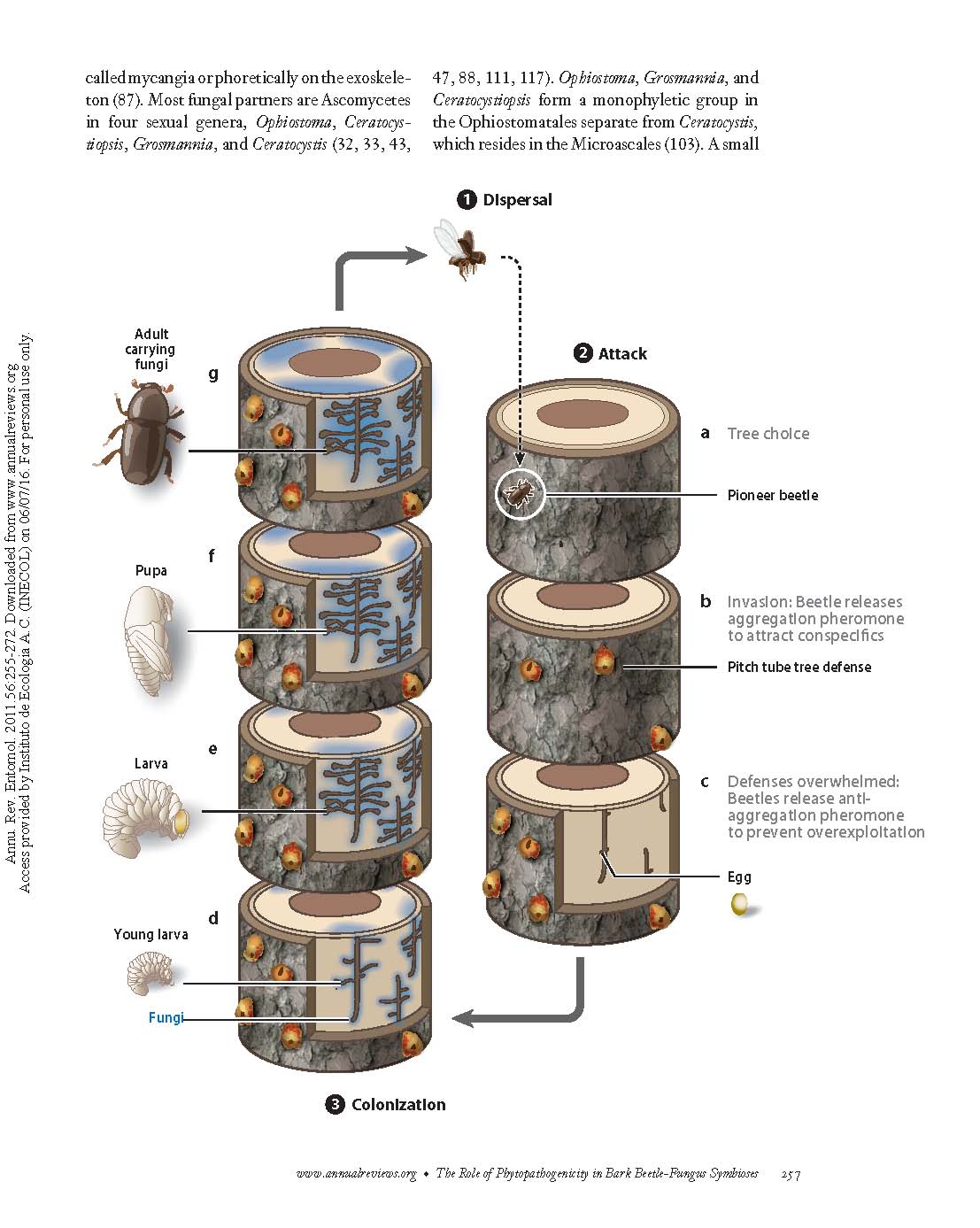


XA


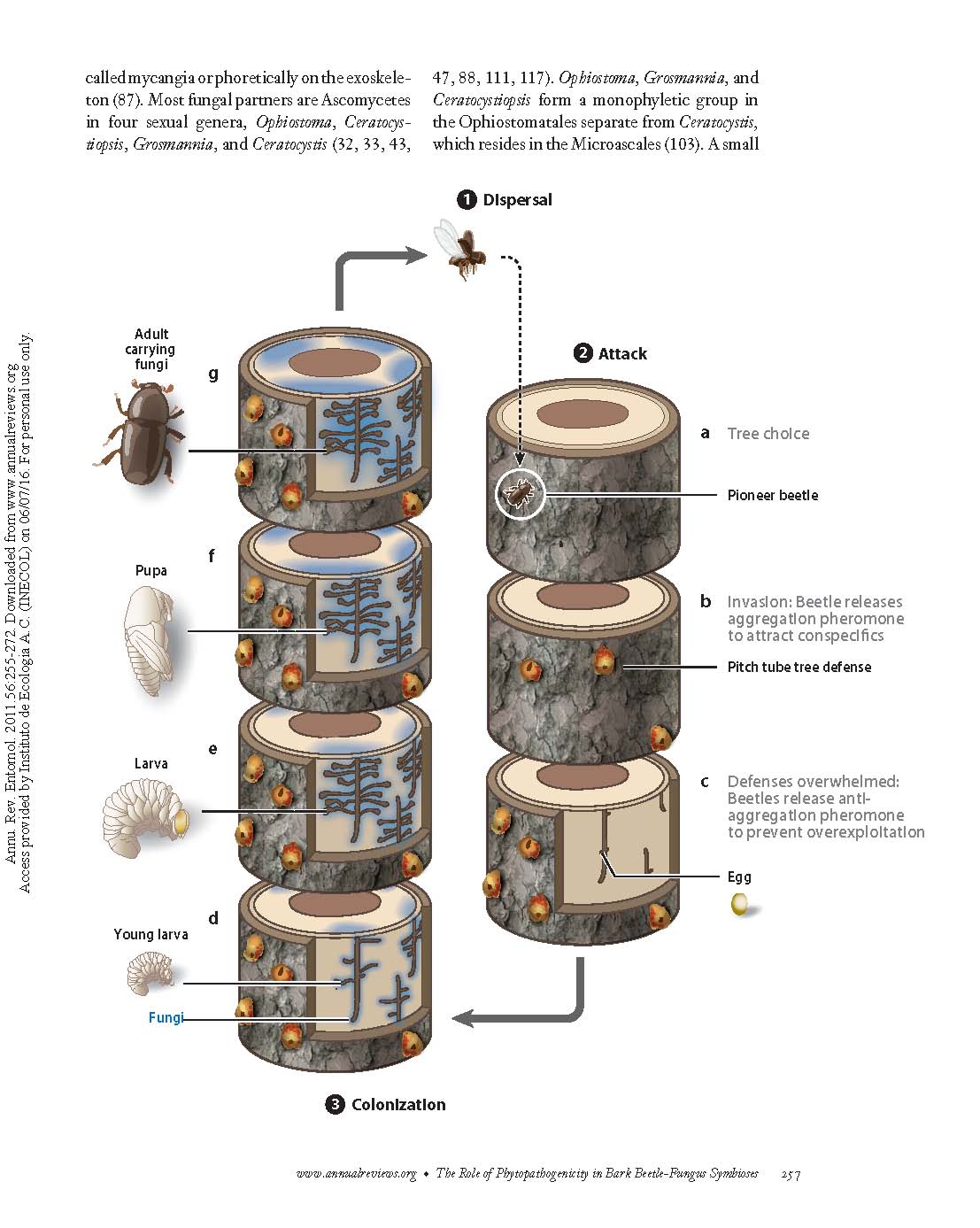


XA

*X. volvulus*


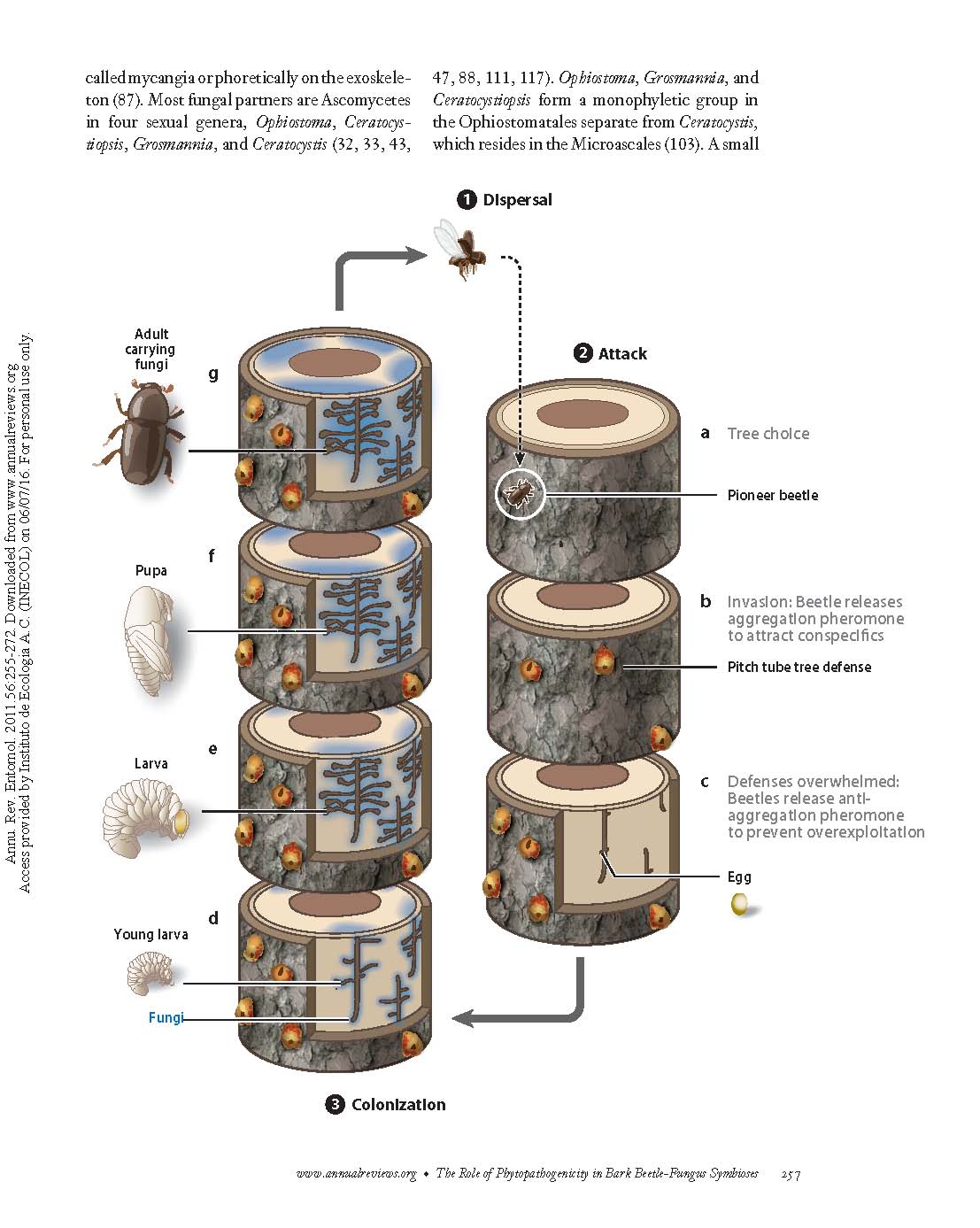


XV


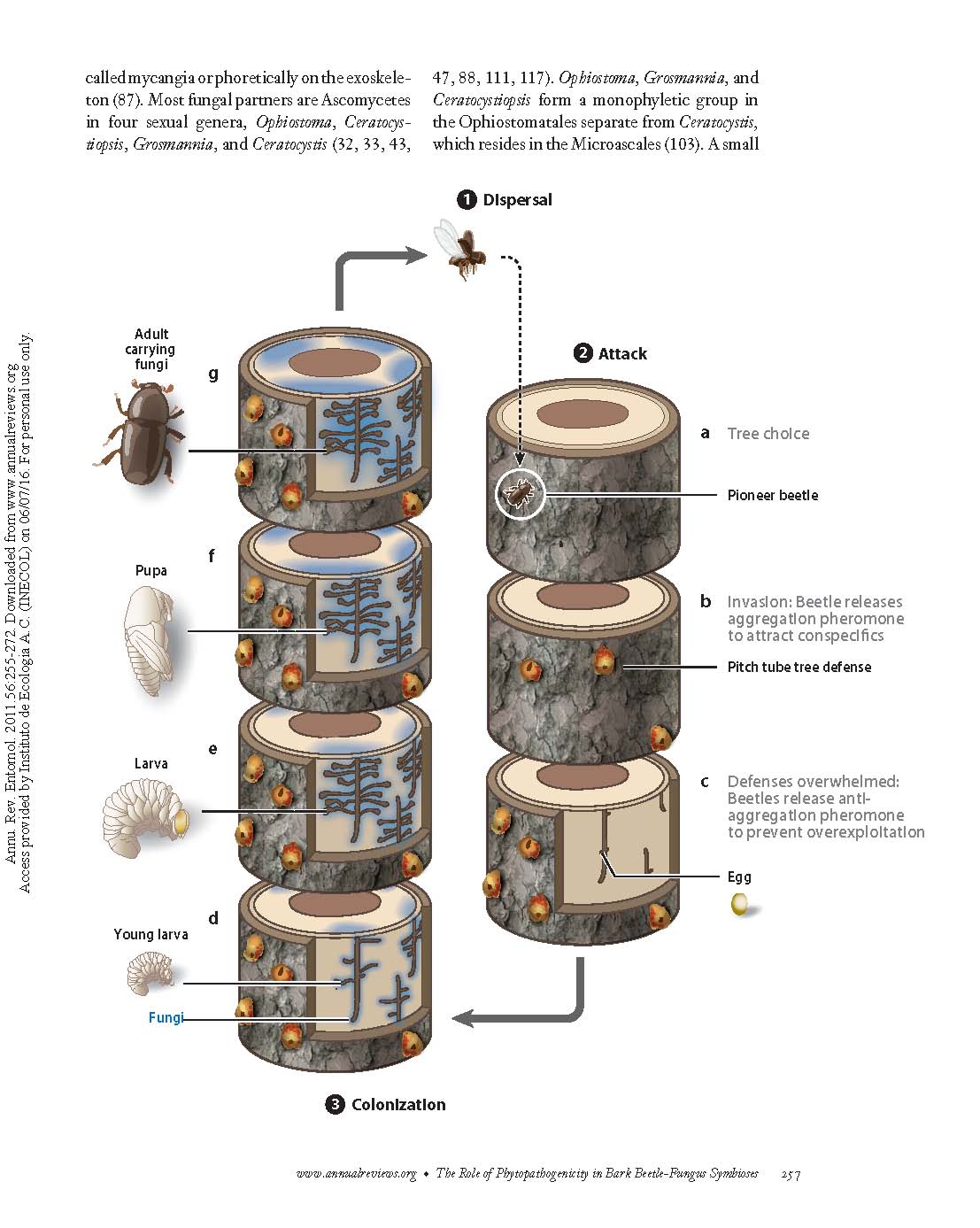


XV

*Persea americana*

**Figure S1: Procurement sampling scheme**. a) *Xyleborus affinis* (XA) and *X. bispinatus* (XB) F1 females were reared on two artificial media based on either *P. schiedeana* or *P. mexicana* sawdust. The microbiome of the F2-offspring females was analyzed. b) The microbiome of *X. affinis* and *X. volvulus* (XV) F1 females that emerged from *B. simaruba* and *P. americana* logs were analyzed.

**(b)**

**(a)**

|  | ■ | X.Aff.CAbdo |
| --- | --- | --- |
|  | ■ | X.Aff.CHead |
|  | ■ | X.Aff.HAbdo |
|  | ■ | X.Aff.HHead |
|  | ■ | X.Bis.CAbdo |
|  | ■ | X.Bis.CHead |
|  | ■ | X.Bis.HAbdo |
|  | ■ | X.Bis.HHead |
|  | ■ | X.Aff |
|  | ■ | X.Vo |


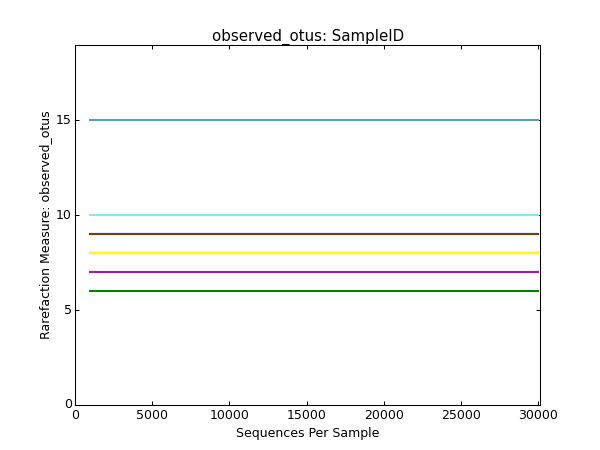

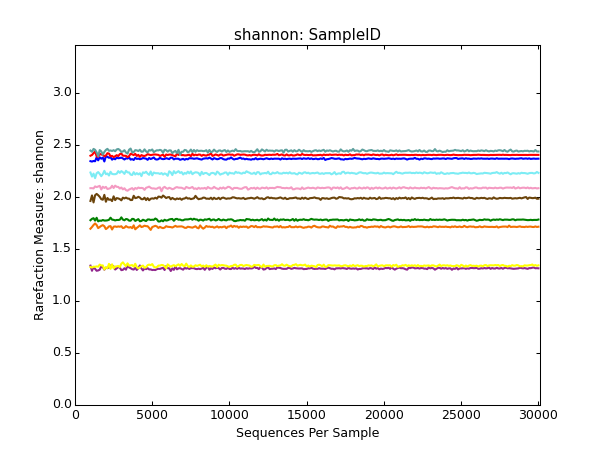


**(c)**

**Legend**


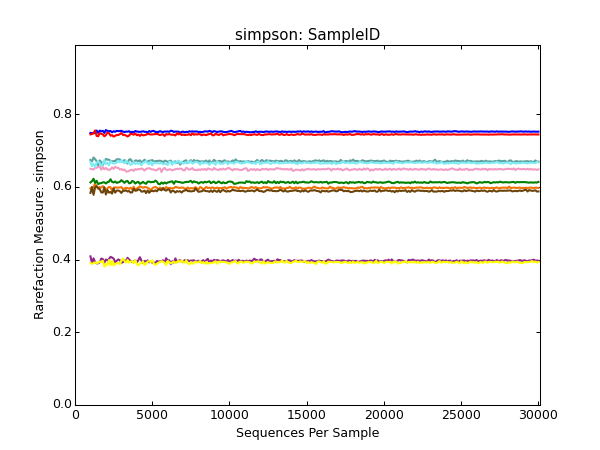


**Figure S2:** Rarefaction curves of pyrosequenced fungal OTUs obtained from the samples of this study after low frequency OTUs (< 0.01%) chimera and low quality reads filtering. a) Number of Observed OTUs, b) Shannon index, c) Simpson index.

**(b)**

**(a)**

**
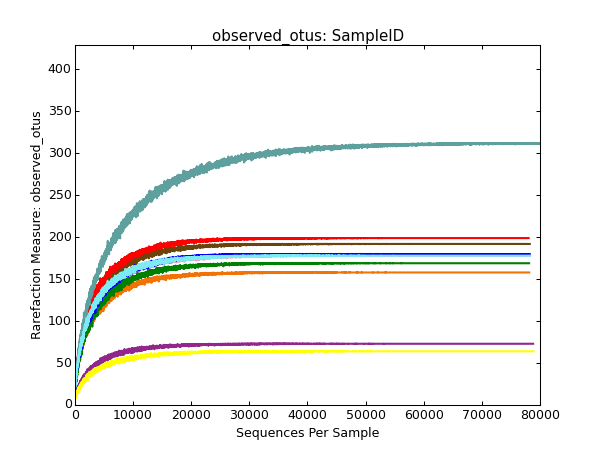

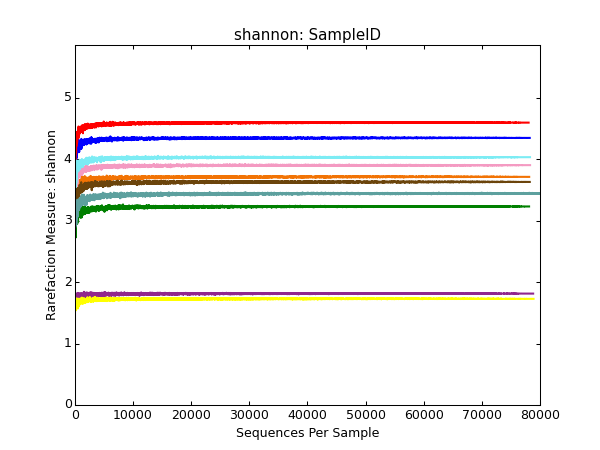
**

**(c)**

|  | ■ | X.Aff.CAbdo |
| --- | --- | --- |
|  | ■ | X.Aff.CHead |
|  | ■ | X.Aff.HAbdo |
|  | ■ | X.Aff.HHead |
|  | ■ | X.Bis.CAbdo |
|  | ■ | X.Bis.CHead |
|  | ■ | X.Bis.HAbdo |
|  | ■ | X.Bis.HHead |
|  | ■ | X.Aff |
|  | ■ | X.Vo |

**
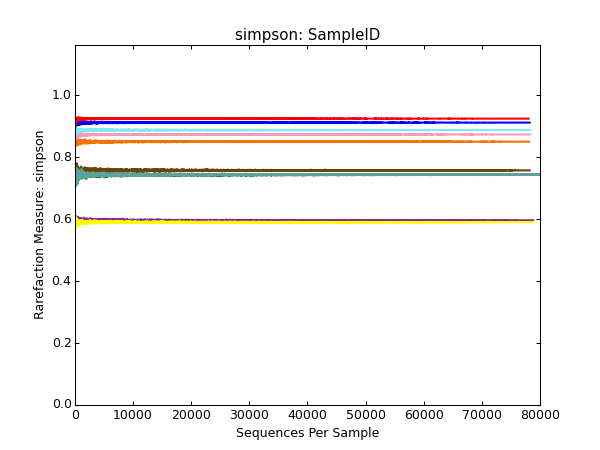
**

**Legend**

**Figure S3:** Rarefaction curves of pyrosequenced bacterial after low frequency OTUs (< 0.01%) chimera and low quality reads filtering. a) Number of Observed OTUs, b) Shannon index, c) Simpson index.

**(a)**

**(b)**

**Figure S4:** Renyi profiles of a) Fungal microbiome, and b) Bacterial microbiome.


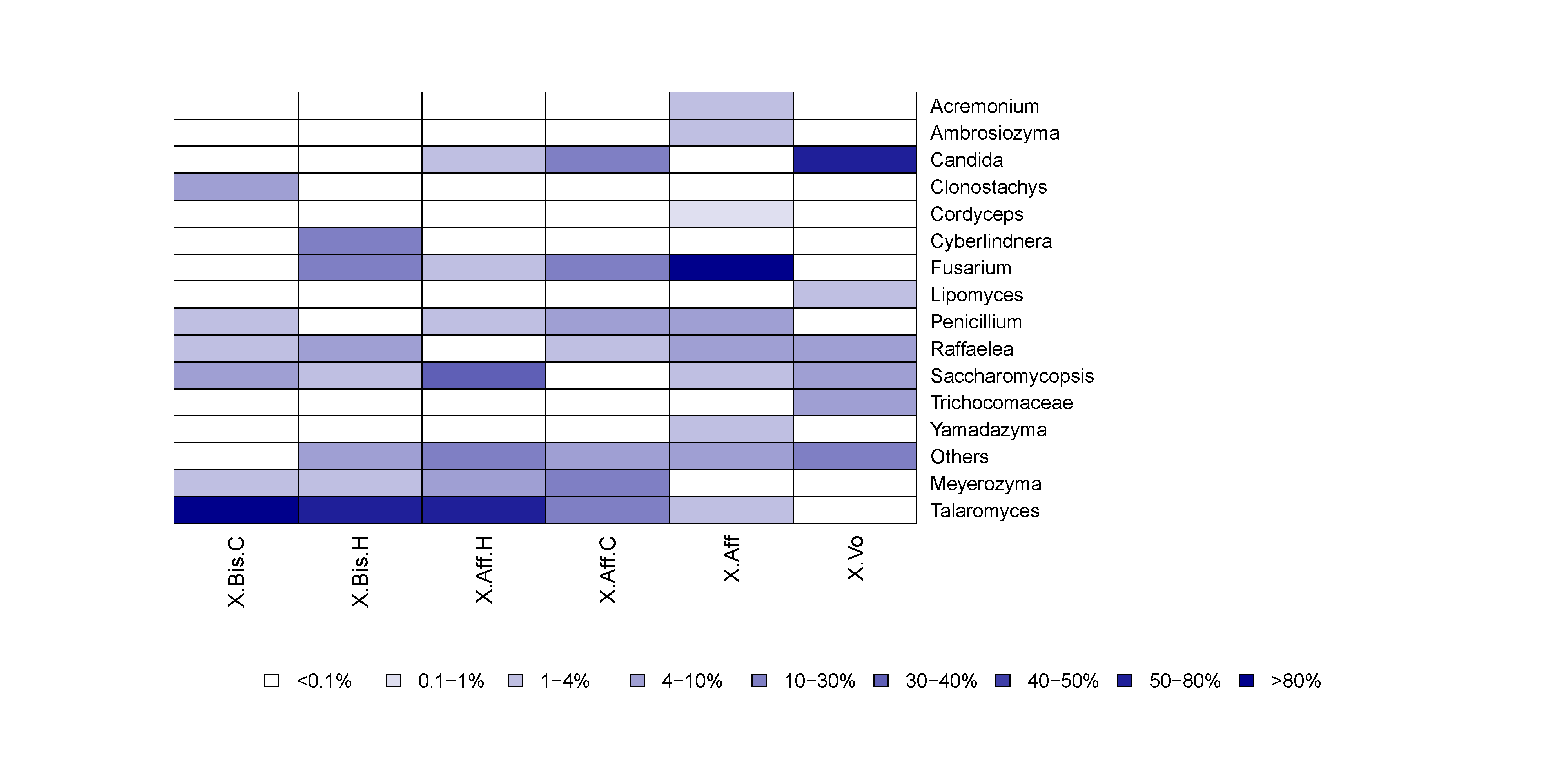


**Figure S5 Relative abundance of the fungal taxa by sample.** The color gradient in the heatmap from white (low) to dark blue (high) represents the relative abundance of the taxa.


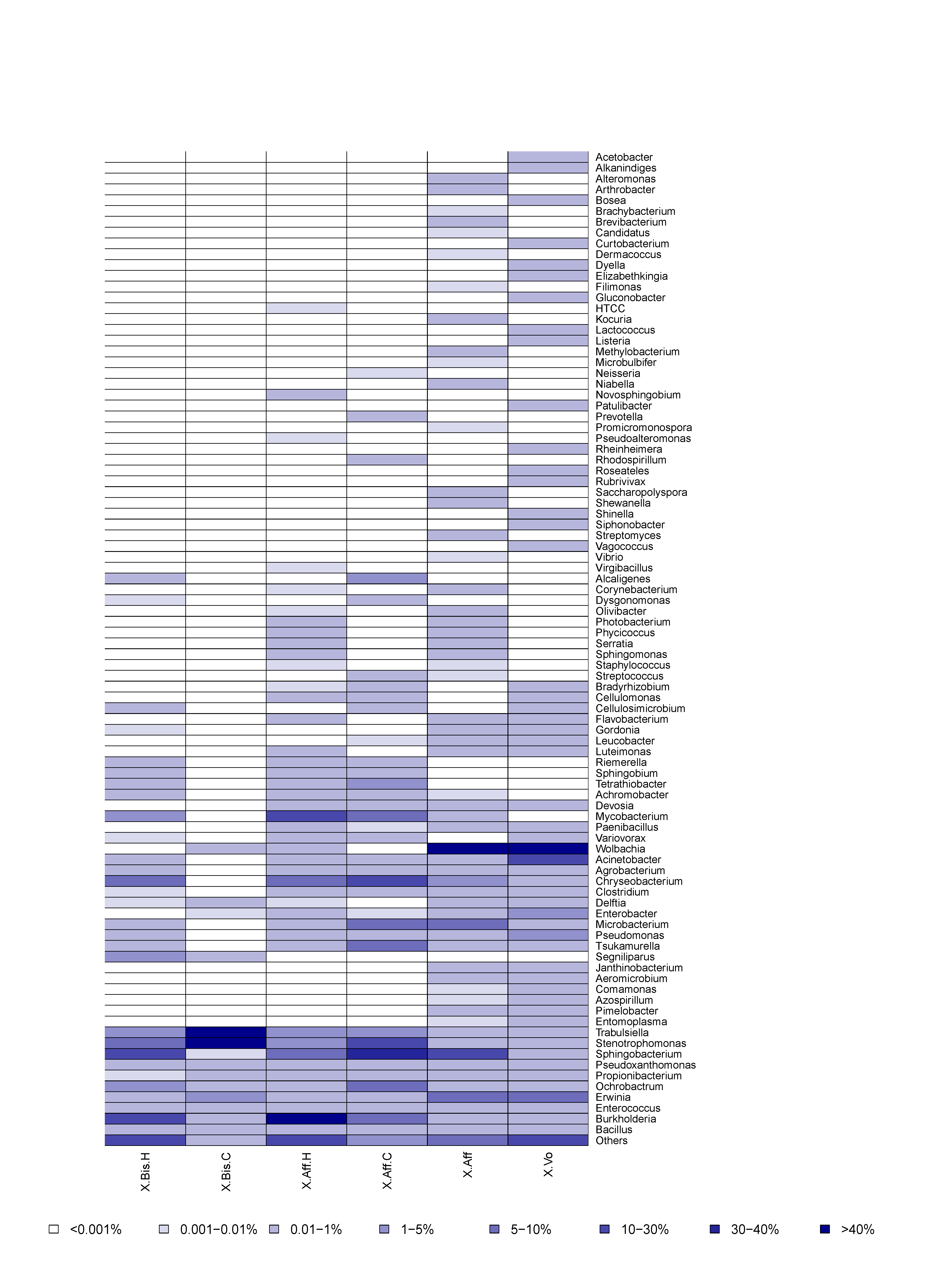


Bacterial Microbiome Core

**Figure S6: Relative abundance of the bacterial genera by sample.** The color gradient in the heatmap from white (low) to dark blue (high) represents the relative abundance of taxa. The genus with orange background is the Bacterial Microbiome Core.

| **Nº ID** | **OTUs; Taxonomy** |  |  |  | **Nº ID** | **OTUs; Taxonomy** | **Nº ID** | **OTUs; Taxonomy** |
| --- | --- | --- | --- | --- | --- | --- | --- | --- |
| 1 | NR.OTU105044; g__*Candida* sp. | | | | 8 | AB003950.1.2138;s_*Clonostachys rosea* | 15 | NCR.OTU23971;g_*Saccharomycopsis* sp. |
| 2 | EU011705.1.1709; g__*Candida* sp. | | | | 9 | EF152417.1.1737;s_*Meyerozyma guilliermondii* | 16 | NR.OTU100069;*g_Saccharomycopsis* sp. |
| 3 | EF027719.1.2165;s_*Fusarium oxysporum* | | | | 10 | AB054883.1.1755;s_*Candida berthetii* | 17 | AF245232.1.1799;s_*Talaromyces purpureogenus* |
| 4 | JPIJ02000041.2177.3960;s_*Fusarium oxysporum* | | | | 11 | EF550459.1.1663;s_*Cyberlindnera fabianii* | 18 | AF245240.1.1799;s_*Talaromyces purpureogenus* |
| 5 | EU710826.1.1658;s_*Fusarium oxysporum* | | | | 12 | KJ909309;g_*Raffaelea* sp. | 19 | FJ216403.1.1528;o_Hypocreales |
| 6 | DQ810190.1.1722;s_*Penicillium chrysogenum* | | | | 13 | JN578863;g_*Raffaelea* sp. |  |  |
| 7 | NCR.OTU49535;s_*Clonostachys rosea* | | | | 14 | AY858666;g_*Raffaelea* sp. |  |  |

**Figure S7: Relative abundance of the fungal OTUs significantly different in the abdomen or the head of *X. bispinatus* and *X. affinis* reared on the different artificial media.** Numbers in the y-axis represent the different fungal OTUS, their taxonomic assignment are listed in the table.

| **Nº ID** | **Genera** |
| --- | --- |
| 1 | *Corynebacterium* |
| 2 | *Phycicoccus* |
| 3 | *Leucobacter* |
| 4 | *Microbacterium* |
| 5 | *Mycobacterium* |
| 6 | *Segniliparus* |
| 7 | *Cellulosimicrobium* |
| 8 | *Propionibacterium* |
| 9 | *Tsukamurella* |
| 10 | *Prevotella* |
| 11 | *Dysgonomonas* |
| 12 | *Chryseobacterium* |
| 13 | *Riemerella* |
| 14 | *Olivibacter* |
| 15 | *Sphingobacterium* |
| 17 | *Paenibacillus* |
| 18 | *Staphylococcus* |
| 19 | *Enterococcus* |
| 20 | *Streptococcus* |
| 21 | *Bradyrhizobium* |
| 22 | *Ochrobactrum* |
| 23 | *Devosia* |
| 24 | *Agrobacterium* |
| 25 | *Wolbachia* |
| 26 | *Novosphingobium* |
| 27 | *Sphingobium* |
| 28 | *Sphingomonas* |
| 29 | *Achromobacter* |
| 30 | *Alcaligenes* |
| 31 | *Tetrathiobacter* |
| 32 | *Burkholderia* |
| 33 | *Delftia* |
| 34 | *Variovorax* |
| 35 | *Neisseria* |
| 36 | *HTCC* |
| 37 | *Enterobacter* |
| 38 | *Erwinia* |
| 39 | *Trabulsiella* |
| 40 | *Acinetobacter* |
| 41 | *Pseudomonas* |
| 42 | *Pseudoalteromonas* |
| 43 | *Photobacterium* |
| 44 | *Luteimonas* |
| 45 | *Pseudoxanthomonas* |
| 46 | *Stenotrophomonas* |

**Figure S8: Relative abundance of bacterial genera differentially present in the abdomen compared to head of the adult females of *X. bispinatus* and *X. affinis* reared on different artificial media.** Numbers in the y-axis represent the different bacterial genera that are listed in the table. The frequencies >0.1% were no represented in the graph.

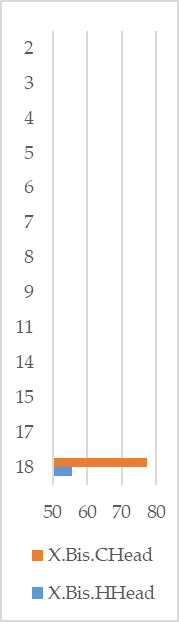

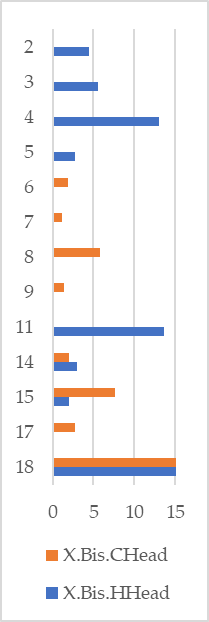


(…)

(…)

| **Nº ID** | **OTUs; Taxonomy** |  |  |  | **Nº ID** | **OTUs; Taxonomy** | **Nº ID** | **OTUs** |
| --- | --- | --- | --- | --- | --- | --- | --- | --- |
| 1 | NR.OTU105044; g__*Candida* sp. | | | | 8 | AB003950.1.2138;s_*Clonostachys rosea* | 15 | NCR.OTU23971;g_*Saccharomycopsis* sp. |
| 2 | EU011705.1.1709; g__*Candida* sp. | | | | 9 | EF152417.1.1737;s_*Meyerozyma guilliermondii* | 16 | NR.OTU100069;*g_Saccharomycopsis* sp. |
| 3 | EF027719.1.2165;s_*Fusarium oxysporum* | | | | 10 | AB054883.1.1755;s_*Candida berthetii* | 17 | AF245232.1.1799;s_*Talaromyces purpureogenus* |
| 4 | JPIJ02000041.2177.3960;s_*Fusarium oxysporum* | | | | 11 | EF550459.1.1663;s_*Cyberlindnera fabianii* | 18 | AF245240.1.1799;s_*Talaromyces purpureogenus* |
| 5 | EU710826.1.1658;s_*Fusarium oxysporum* | | | | 12 | KJ909309;g_*Raffaelea* sp. | 19 | FJ216403.1.1528;o_Hypocreales |
| 6 | DQ810190.1.1722;s_*Penicillium chrysogenum* | | | | 13 | JN578863;g_*Raffaelea* sp. |  |  |
| 7 | NCR.OTU49535;s_*Clonostachys rosea* | | | | 14 | AY858666;g_*Raffaelea* sp. |  |  |

**Figure S9: Relative abundance of the fungal OTUs differentially present in the abdomen and head of *X. affinis* and *X. bispinatus* comparing rearing conditions.** Numbers in the y-axis represent the different fungal OTUS, their taxonomic assignment are listed in the table. The frequencies >0.1% were no represented in the graph.

**Figure S10: Relative abundance of the bacterial genera significantly different in the abdomen and the head of *X. affinis* and *X. bispinatus* comparing rearing conditions.** Numbers in the y-axis represent the different bacterial genera listed in the table. The frequencies >1% were no represented in the graph.

*X. affinis* wild

*X. volvulus* wild

**Figure S11: Fold changes (GFOLD (0.01)) of the OTUs with significant larger abundance in wild beetles compared to the lab reared beetles.** *X. affinis* reared in *P. schiedeana* sawdust (Aff.C); *X. affinis* reared in *P. mexicana* sawdust (Aff.H); *X. bispinatus* reared in *P. schiedeana* sawdust (Bis.C); Bis.H: *X. bispinatus* fed in *P. mexicana* sawdust media. Numbers in the y-axis represent the different bacterial OTUS, their taxonomic assignment are listed in the table. See table S12.

**Figure S12: PCoA plot of the samples beta diversity of Weighted Unifrac of:** a) Fungal OTUs, b) Bacterial OTUs.


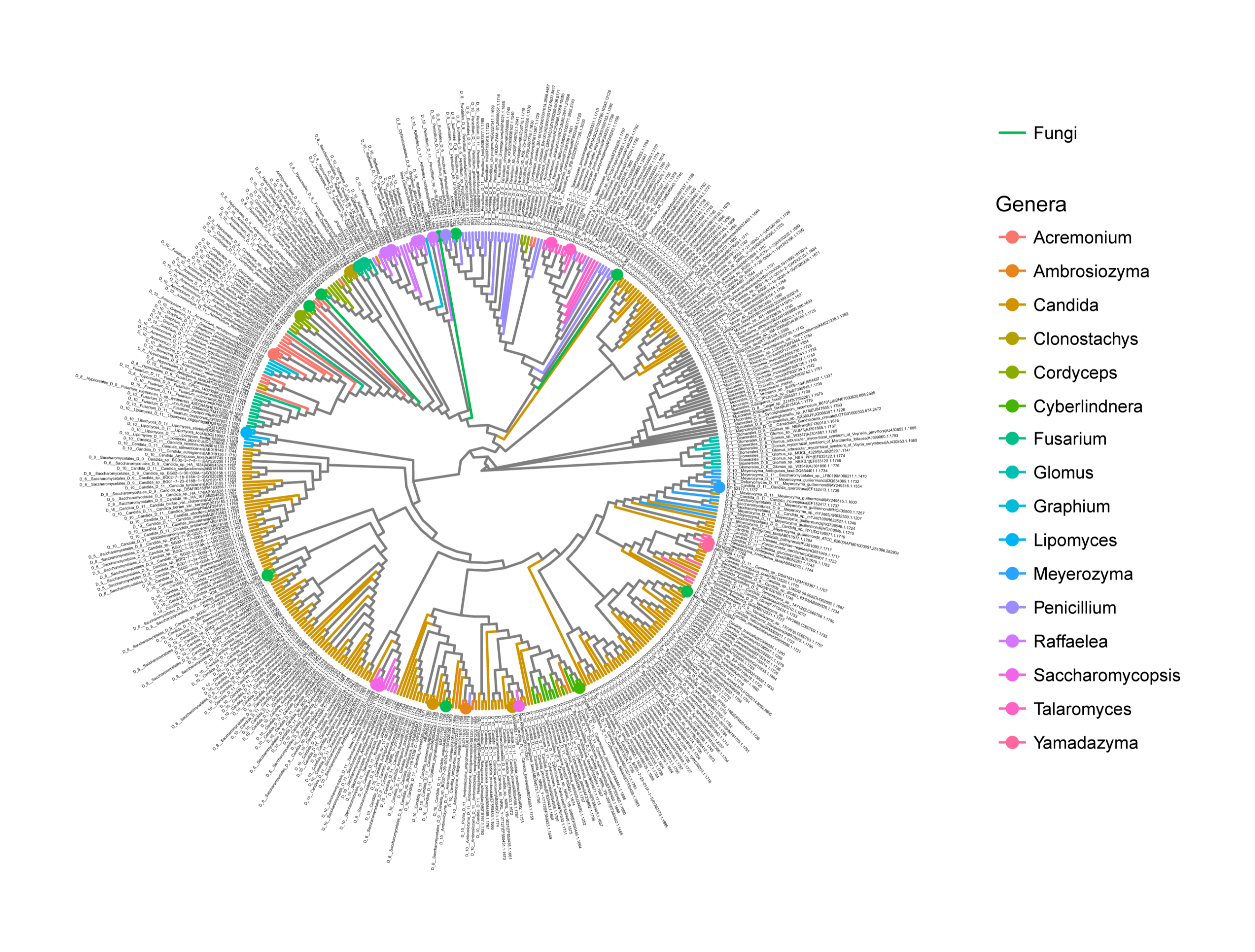


**Figure S13.** Phylogenetic tree of the OTUs and reference sequences: Branch colors represent each fungi genus, circles within phylogeny represents OTU sequences; color circles represent the taxonomical affiliation based on OTU clustering analysis. OTUs without taxonomic annotation were designated as fungi.


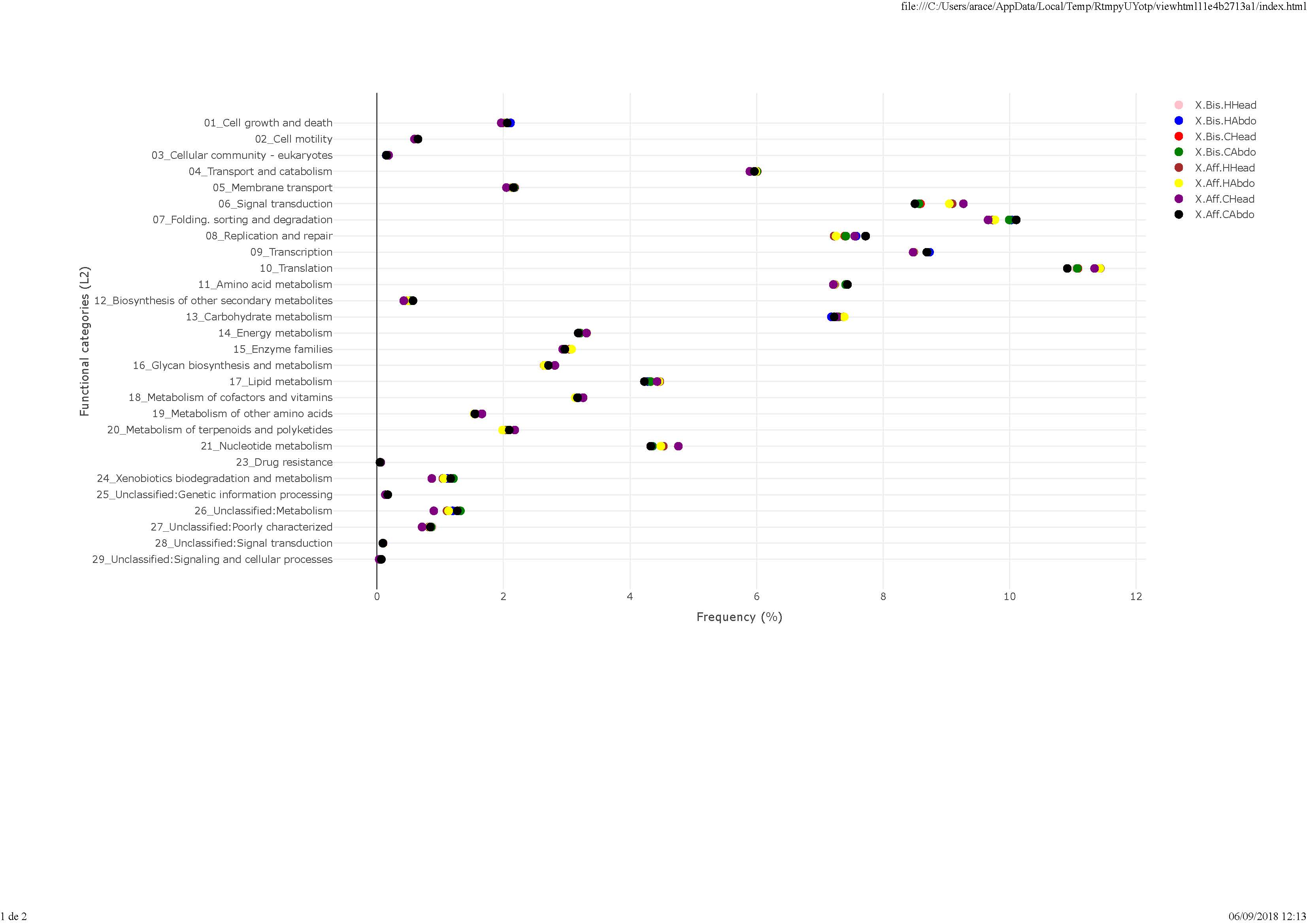


**Figure S14.** **Dot-plot of the functional categories frequencies of the fungal microbiome of the laboratory-reared beetles.** The functional categories were based on KEGG database at level 2.


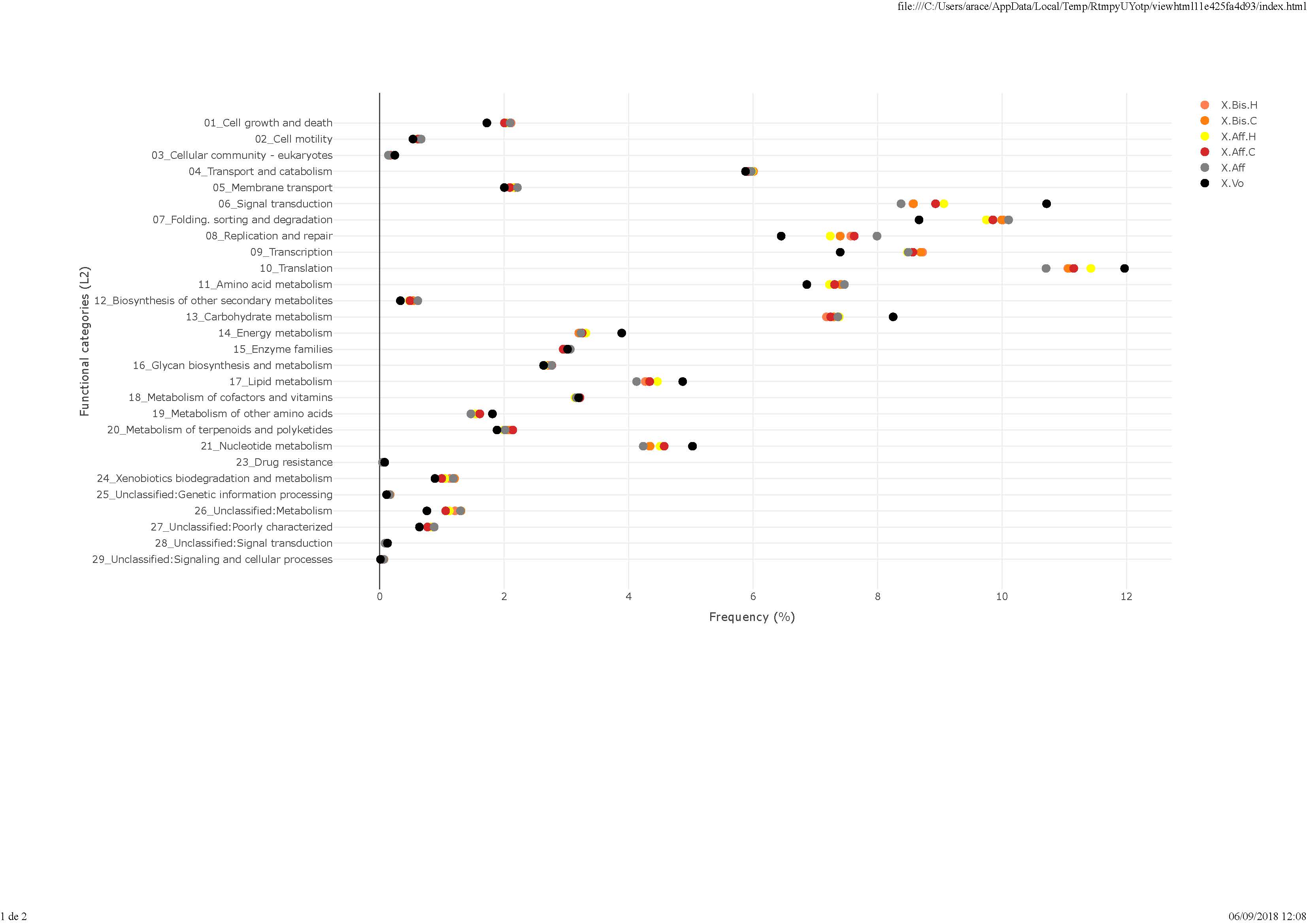


**Figure S15. Dot-plot of the functional categories frequencies of the fungal microbiome.** The functional categories were based on KEGG database at level 2.


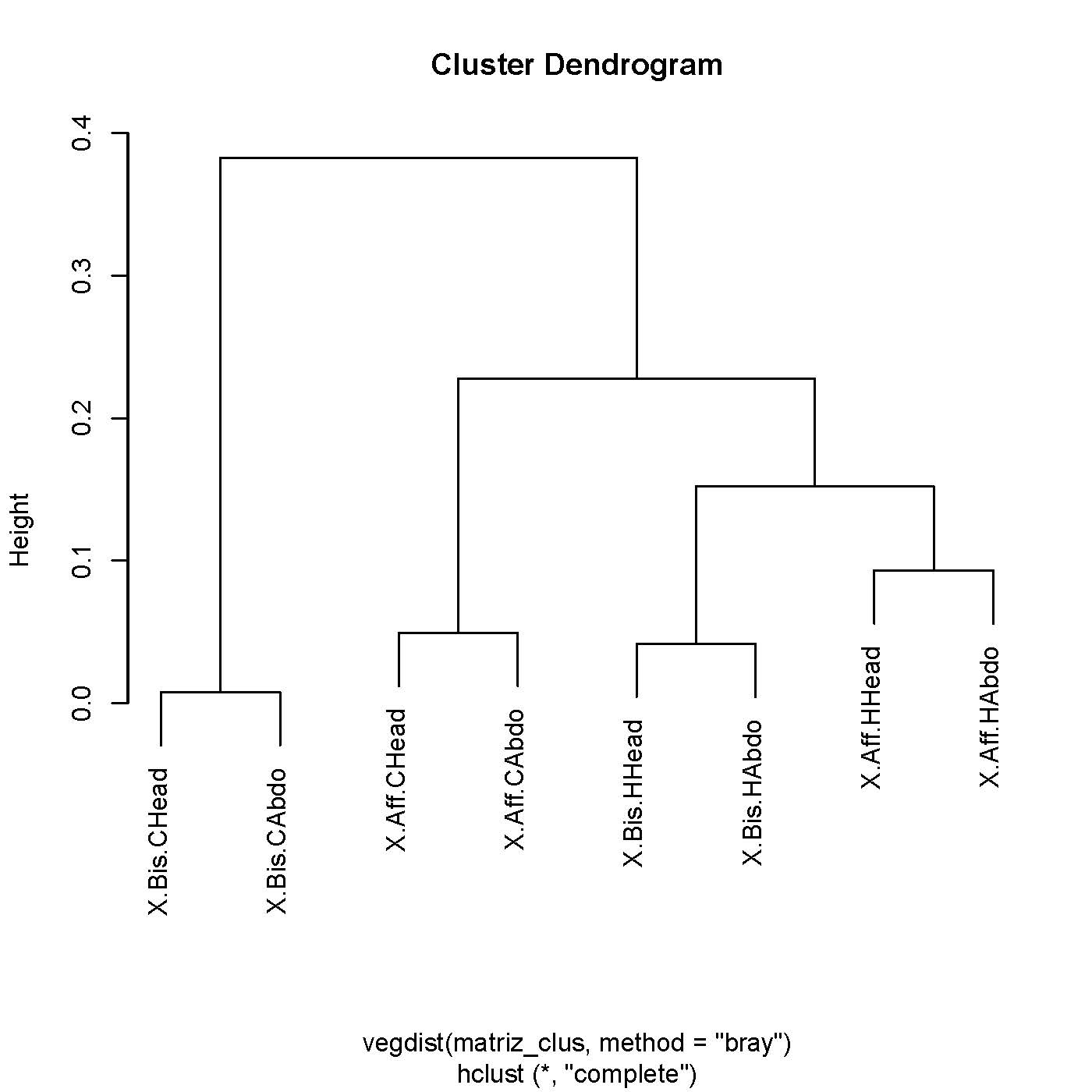


**Figure S16:** Cluster dendrogram of dissimilarity matrix of the abundance of KOs: Head of *X. bispinatus* reared on *P. mexicana* sawdust media (X.Bis. HHead); Abdomen of *X. bispinatus* reared on *P. mexicana* media (X.Bis.HAbdo); Head of *X. bispinatus* reared on *P. schiedeana* media (X.Bis.CHead); Abdomen of *X. bispinatus* reared on *P. schiedeana* media (X.Bis.CAbdo); Head of *X. affinis* reared on *P. mexicana* sawdust media (X.Aff.HHead); Abdomen of *X. affinis* reared on *P. mexicana* media (X.Aff.HAbdo); Head of *X. affinis* reared on *P. schiedeana* sawdust media (X.Aff.CHead)*;* Abdomen of *X. bispinatus* reared on *P. schiedeana* media (X.Aff.CAbdo).

| **Nº ID** | **Functional categories** |
| --- | --- |
| 1 | Cell growth and death |
| 2 | Cell motility |
| 3 | Cellular community - prokaryotes |
| 4 | Cellular Processes |
| 5 | Transport and catabolism |
| 6 | Membrane transport |
| 7 | Signal transduction |
| 8 | Folding, sorting, and degradation |
| 9 | Transcription |
| 10 | Translation |
| 11 | Replication and repair |
| 12 | Drug resistance |
| 13 | Infectious diseases |
| 14 | Amino acid metabolism |
| 15 | Biosynthesis of other secondary metabolites |
| 16 | Carbohydrate metabolism |
| 17 | Energy metabolism |
| 18 | Enzyme families |
| 19 | Glycan biosynthesis and metabolism |
| 20 | Lipid metabolism |
| 21 | Metabolism of cofactors and vitamins |
| 22 | Metabolism of other amino acids |
| 23 | Metabolism of terpenoids and polyketides |
| 24 | Nucleotide metabolism |
| 25 | Xenobiotics biodegradation and metabolism |
| 26 | Unclassified-Cellular processes and signaling |
| 27 | Unclassified-Genetic information processing |
| 28 | Unclassified-Metabolism |
| 29 | Poorly characterized |

**Figure S17: Relative frequency of the functional** **categories with differences in abundance between head and abdomen of the beetles reared under lab condition.** Numbers in the y-axis represent the different functional categories listed in the table.

| **Nº ID** | **Functional categories** |
| --- | --- |
| 1 | Cell growth and death |
| 2 | Cell motility |
| 3 | Cellular community - prokaryotes |
| 4 | Cellular Processes |
| 5 | Transport and catabolism |
| 6 | Membrane transport |
| 7 | Signal transduction |
| 8 | Folding, sorting, and degradation |
| 9 | Transcription |
| 10 | Translation |
| 11 | Replication and repair |
| 12 | Drug resistance |
| 13 | Infectious diseases |
| 14 | Amino acid metabolism |
| 15 | Biosynthesis of other secondary metabolites |
| 16 | Carbohydrate metabolism |
| 17 | Energy metabolism |
| 18 | Enzyme families |
| 19 | Glycan biosynthesis and metabolism |
| 20 | Lipid metabolism |
| 21 | Metabolism of cofactors and vitamins |
| 22 | Metabolism of other amino acids |
| 23 | Metabolism of terpenoids and polyketides |
| 24 | Nucleotide metabolism |
| 25 | Xenobiotics biodegradation and metabolism |
| 26 | Unclassified-Cellular processes and signaling |
| 27 | Unclassified-Genetic information processing |
| 28 | Unclassified-Metabolism |
| 29 | Poorly characterized |

**Figure S18: Relative abundance of the functional** **categories with differences in abundance between *X. bispinatus* and *X. affinis* reared under lab conditions.** Numbers in the y-axis represent the different functional categories listed in the table.


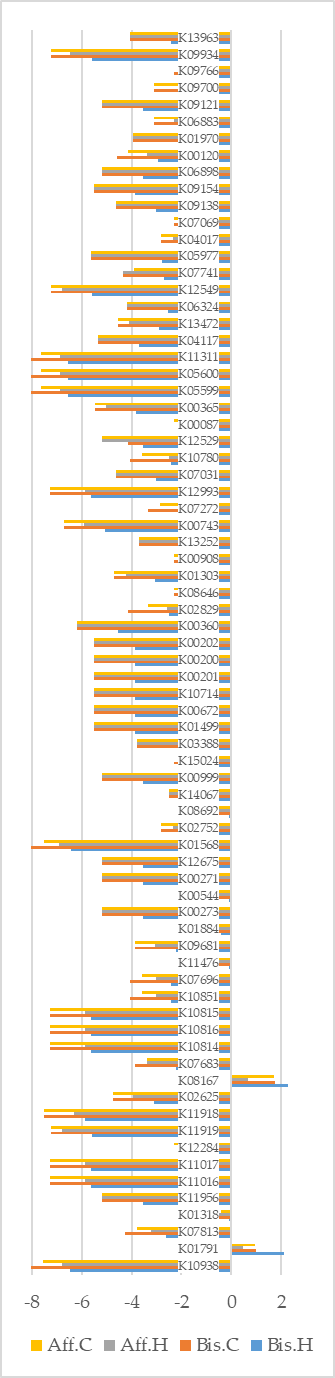

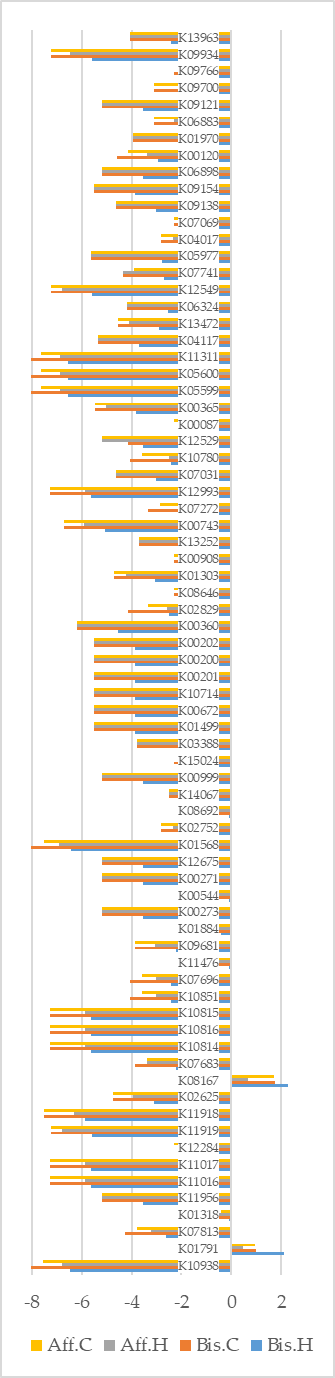


*X. affinis* wild

*X. volvulus* wild

**Fig. S19. Change fold significate different of the KO’s between wild and reared lab condition beetles.** *X. affinis* reared on *P. schiedeana* medium (Aff.C); *X. affinis* reared on *P. mexicana* medium (Aff.H); *X. bispinatus* reared on *P. schiedeana* medium (Bis.C); *X. bispinatus* reared on *P. mexicana* medium (Bis.H).

**Table S16. Relative frequencies of significate different of the KO’s between wild and reared lab condition beetles.**

|  | Bis.H | Bis.C | Aff.H | Aff.C | X.Vo | X.Aff |
| --- | --- | --- | --- | --- | --- | --- |
| K01884 | 0 | 0 | 0,01025352 | 0 | 0,05905512 | 0,03422899 |
| K07069 | 0,00213566 | 0 | 0,02819718 | 0,00334158 | 0,06889764 | 0,04278624 |
| K11476 | 0 | 0 | 0,01794366 | 0,00501236 | 0,0492126 | 0,07273661 |
| K13963 | 0 | 0 | 0 | 0 | 0,22637795 | 0,04278624 |
| K03388 | 0 | 0 | 0 | 0 | 0,18700787 | 0,05990074 |
| K09681 | 0 | 0 | 0,0128169 | 0,00167079 | 0,19685039 | 0,03850762 |
| K01970 | 0,01067828 | 0 | 0,00512676 | 0 | 0,20669291 | 0,02995037 |
| K09766 | 0,00213566 | 0 | 0,0256338 | 0,00501236 | 0,06889764 | 0,06417936 |
| K15024 | 0,00213566 | 0 | 0,02819718 | 0,00501236 | 0,06889764 | 0,06845798 |
| K07683 | 0 | 0 | 0,00769014 | 0,00501236 | 0,19685039 | 0,05134349 |
| K08646 | 0 | 0,00193056 | 0,0128169 | 0 | 0,06889764 | 0,1069656 |
| K00908 | 0 | 0,00193056 | 0,0128169 | 0 | 0,06889764 | 0,1069656 |
| K13472 | 0 | 0 | 0,00769014 | 0 | 0,31496063 | 0,02567174 |
| K09138 | 0 | 0 | 0 | 0 | 0,33464567 | 0,03850762 |
| K07741 | 0 | 0 | 0 | 0,00501236 | 0,27559055 | 0,05134349 |
| K00544 | 0 | 0 | 0,00769014 | 0 | 0,0492126 | 0,16258771 |
| K02625 | 0 | 0 | 0,0128169 | 0 | 0,35433071 | 0,02567174 |
| K10851 | 0 | 0 | 0,01794366 | 0,00501236 | 0,22637795 | 0,05990074 |
| K07813 | 0 | 0 | 0,01794366 | 0,00668315 | 0,25590551 | 0,05990074 |
| K07696 | 0 | 0 | 0,01794366 | 0,00668315 | 0,22637795 | 0,07701523 |
| K12284 | 0 | 0,00514817 | 0,01794366 | 0 | 0,06889764 | 0,14547322 |
| K00120 | 0,00427131 | 0 | 0,02050704 | 0,00668315 | 0,32480315 | 0,05134349 |
| K06883 | 0 | 0,00193056 | 0,0128169 | 0 | 0,11811024 | 0,17114496 |
| K00087 | 0,00427131 | 0,01287043 | 0,0128169 | 0 | 0,06889764 | 0,11124422 |
| K10780 | 0,00640697 | 0 | 0,02819718 | 0,00668315 | 0,22637795 | 0,09412973 |
| K02829 | 0,00213566 | 0 | 0,02819718 | 0,00835394 | 0,23622047 | 0,09840835 |
| K06324 | 0 | 0 | 0,00256338 | 0 | 0,24606299 | 0,19253808 |
| K00999 | 0 | 0 | 0 | 0 | 0,48228346 | 0,11124422 |
| K11956 | 0 | 0 | 0 | 0 | 0,48228346 | 0,11124422 |
| K12675 | 0 | 0 | 0 | 0 | 0,48228346 | 0,11980147 |
| K07031 | 0 | 0 | 0 | 0 | 0,33464567 | 0,20965258 |
| K13252 | 0 | 0 | 0 | 0 | 0,17716535 | 0,28238918 |
| K09700 | 0 | 0,00193056 | 0,02050704 | 0 | 0,11811024 | 0,27811056 |
| K04117 | 0 | 0 | 0 | 0 | 0,54133858 | 0,14547322 |
| K12529 | 0,00427131 | 0,01029634 | 0 | 0 | 0,48228346 | 0,11124422 |
| K08692 | 0 | 0 | 0 | 0 | 0,0492126 | 0,43214102 |
| K14067 | 0 | 0 | 0 | 0 | 0,07874016 | 0,45781277 |
| K01303 | 0 | 0 | 0,01025352 | 0 | 0,34448819 | 0,35084717 |
| K00360 | 0 | 0 | 0 | 0 | 0,96456693 | 0,22248845 |
| K01499 | 0 | 0 | 0 | 0 | 0,61023622 | 0,44069827 |
| K00672 | 0 | 0 | 0 | 0 | 0,61023622 | 0,44069827 |
| K10714 | 0 | 0 | 0 | 0 | 0,61023622 | 0,44069827 |
| K00201 | 0 | 0 | 0 | 0 | 0,61023622 | 0,44069827 |
| K00200 | 0 | 0 | 0 | 0 | 0,61023622 | 0,44069827 |
| K00202 | 0 | 0 | 0 | 0 | 0,61023622 | 0,44069827 |
| K09154 | 0 | 0 | 0 | 0 | 0,61023622 | 0,44069827 |
| K00743 | 0 | 0,00193056 | 0,0128169 | 0 | 1,37795276 | 0,11980147 |
| K00271 | 0 | 0 | 0 | 0 | 0,48228346 | 0,6246791 |
| K12549 | 0 | 0 | 0,01025352 | 0,00167079 | 2,00787402 | 0,04278624 |
| K07272 | 0 | 0 | 0,02050704 | 0,00501236 | 0,13779528 | 0,83005305 |
| K11919 | 0 | 0 | 0,01025352 | 0,00167079 | 2,00787402 | 0,04278624 |
| K04017 | 0 | 0,00257409 | 0,01025352 | 0 | 0,0984252 | 0,88139654 |
| K09934 | 0 | 0,00064352 | 0,0128169 | 0,00167079 | 2,00787402 | 0,05990074 |
| K02752 | 0 | 0,00321761 | 0,01025352 | 0 | 0,0984252 | 0,88139654 |
| K12993 | 0 | 0 | 0,02307042 | 0,00167079 | 2,02755906 | 0,08557248 |
| K11016 | 0 | 0 | 0,02307042 | 0,00167079 | 2,02755906 | 0,08557248 |
| K10814 | 0 | 0 | 0,02307042 | 0,00167079 | 2,02755906 | 0,08557248 |
| K10816 | 0 | 0 | 0,02307042 | 0,00167079 | 2,02755906 | 0,08557248 |
| K10815 | 0 | 0 | 0,02307042 | 0,00167079 | 2,02755906 | 0,08557248 |
| K11017 | 0 | 0 | 0,02307042 | 0,00167079 | 2,02755906 | 0,12835872 |
| K00273 | 0 | 0 | 0 | 0 | 0,48228346 | 1,03970563 |
| K11918 | 0 | 0,00193056 | 0,02050704 | 0,00167079 | 2,42125984 | 0,15830909 |
| K01318 | 0 | 0 | 0 | 0,01169552 | 0,0492126 | 1,51035427 |
| K09121 | 0 | 0 | 0 | 0,00167079 | 0,48228346 | 1,57453363 |
| K06898 | 0 | 0 | 0 | 0,00167079 | 0,48228346 | 1,59164813 |
| K01568 | 0,00854263 | 0 | 0,03332393 | 0,01002473 | 4,92125984 | 0,09412973 |
| K05599 | 0,00854263 | 0 | 0,04357745 | 0,01002473 | 5,35433071 | 0,22676707 |
| K05600 | 0,00854263 | 0 | 0,04357745 | 0,01002473 | 5,35433071 | 0,22676707 |
| K11311 | 0,00854263 | 0 | 0,04357745 | 0,01002473 | 5,35433071 | 0,22676707 |
| K00365 | 0 | 0 | 0,01025352 | 0,00167079 | 0,59055118 | 2,44737292 |
| K10938 | 0,00854263 | 0 | 0,03588731 | 0,01002473 | 5 | 0,46637002 |
| K05977 | 0,01922091 | 0 | 0,00256338 | 0 | 0,65944882 | 4,4754407 |
| K08167 | 39,3815138 | 49,7023714 | 39,6785522 | 49,6224019 | 12,765748 | 37,1940784 |
| K01791 | 60,5181104 | 50,2512951 | 59,465279 | 50,2188732 | 22,0964567 | 36,7277084 |

**Table S17. Functional categories of the KEGG Orthology (KO’s) with significate abundance difference between wild and reared lab conditions beetles.**

| **KO's** | **Metabolic category** | |
| --- | --- | --- |
| K10938 | Biofilm formation - Vibrio cholerae | |
| K01791 | Biofilm formation - Vibrio cholerae; Carbohydrate Amino sugar and nucleotide sugar metabolism; Glycan biosynthesis and Lipopolysaccharide biosynthesis proteins | |
| K07813 | Quorum sensing; Two-component system; Peptidases | |
| K01318 | Quorum sensing; Peptidases | |
| K11956 | ABC transporters; Transporters | |
| K11016 | Bacterial secretion system; Secretion system; Bacterial toxins | |
| K11017 | Bacterial secretion system; Secretion system; Bacterial toxins | |
| K12284 | Secretion system | |
| K11919 | Secretion system | |
| K11918 | Secretion system | |
| K02625 | Transporters |  |
| K08167 | Transporters; Antimicrobial resistance genes | |
| K07683 | Two-component system | |
| K10814 | Bacterial toxins;Cyanoamino acid metabolism | |
| K10816 | Bacterial toxins;Cyanoamino acid metabolism | |
| K10815 | Bacterial toxins;Cyanoamino acid metabolism | |
| K10851 | Two-component system | |
| K07696 | Two-component system | |
| K11476 | Transcription factors | |
| K09681 | Transcription factors | |
| K01884 | Aminoacyl-tRNA biosynthesis | |
| K00273 | Amino acid Arginine and proline metabolism;Amino acid Glycine, serine and threonine metabolism;Penicillin and cephalosporin biosynthesis;D-Arginine and D-ornithine metabolism | |
| K00544 | Amino acid Cysteine and methionine metabolism; Amino acid Glycine, serine and threonine metabolism | |
| K00271 | Amino acid Valine, leucine and isoleucine degradation | |
| K12675 | Clavulanic acid biosynthesis | |
| K01568 | Carbohydrate Glycolysis / Gluconeogenesis | |
| K02752 | Carbohydrate Glycolysis / Gluconeogenesis; Phosphotransferase system (PTS) | |
| K08692 | Carbohydrate Glyoxylate and dicarboxylate metabolism; Energy Methane metabolism | |
| K14067 | Carbohydrate Glyoxylate and dicarboxylate metabolism; Energy Methane metabolism | |
| K00999 | Carbohydrate Inositol phosphate metabolism; Lipid Glycerophospholipid metabolism | |
| K15024 | Carbohydrate Pyruvate metabolism; Carbohydrate Propanoate metabolism; Energy Carbon fixation pathways in prokaryotes; Taurine and hypotaurine | |
| K03388 | Energy Methane metabolism | |
| K01499 | Energy Methane metabolism | |
| K00672 | Energy Methane metabolism | |
| K10714 | Energy Methane metabolism | |
| K00201 | Energy Methane metabolism | |
| K00200 | Energy Methane metabolism | |
| K00202 | Energy Methane metabolism | |
| K00360 | Energy Nitrogen metabolism | |
| K02829 | Energy Oxidative phosphorylation | |
| K08646 | Peptidases |  |
| K01303 | Peptidases |  |
| K00908 | Transferases |  |
| K13252 | Transferases |  |
| K00743 | Glycan biosynthesis and Glycosyltransferases | |
| K07272 | Glycan biosynthesis and Glycosyltransferases; Glycan biosynthesis and Lipopolysaccharide biosynthesis proteins | |
| K12993 | Glycan biosynthesis and Glycosyltransferases; Glycan biosynthesis and Lipopolysaccharide biosynthesis proteins | |
| K07031 | Glycan biosynthesis and Lipopolysaccharide biosynthesis | |
| K10780 | Lipid Fatty acid biosynthesis | |
| K12529 | Selenocompound metabolism | |
| K00087 | Nucleotide Purine metabolism | |
| K00365 | Nucleotide Purine metabolism; Caffeine metabolism | |
| K05599 | Xenobiotics biodegradation and Aminobenzoate degradation | |
| K05600 | Xenobiotics biodegradation and Aminobenzoate degradation | |
| K11311 | Xenobiotics biodegradation and Aminobenzoate degradation | |
| K04117 | Xenobiotics biodegradation and Benzoate degradation | |
| K13472 | Plant-pathogen interaction | |
| K06324 | Cell growth |  |
| K12549 | Cell motility and secretion | |
| K07741 | Transcription related proteins | |
| K05977 | Biosynthesis and biodegradation of secondary metabolites | |
| K04017 | Energy metabolism | |
| K07069 | Function unknown | |
| K09138 | Function unknown | |
| K09154 | Function unknown | |
| K06898 | Function unknown | |
| K00120 | Function unknown | |
| K01970 | Function unknown | |
| K06883 | Function unknown | |
| K09121 | Function unknown | |
| K09700 | Function unknown | |
| K09766 | Function unknown | |
| K09934 | Function unknown | |
| K13963 | Function unknown | |
